# OASL phase condensation induces amyloid-like fibrillation of RIPK3 to promote virus-induced necroptosis

**DOI:** 10.1038/s41556-022-01039-y

**Published:** 2023-01-05

**Authors:** Shin-Ae Lee, Lin-Chun Chang, WooRam Jung, James W. Bowman, Dokyun Kim, Weiqiang Chen, Suan-Sin Foo, Youn Jung Choi, Un Yung Choi, Anna Bowling, Ji-Seung Yoo, Jae U. Jung

**Affiliations:** 1 Department of Cancer Biology, Infection Biology Program, and Global Center for Pathogen Research and Human Health, Lerner Research Institute, Cleveland Clinic, Cleveland, OH USA; 2 Department of Molecular Microbiology and Immunology, Keck School of Medicine, University of Southern California, Los Angeles, CA USA; 3 Immunology Program of the Sloan Kettering Institute, Memorial Sloan Kettering Cancer Center, New York, NY USA; 4 Center for Study of Emerging and Re-emerging Viruses, Korea Virus Research Institute, Institute for Basic Science, Daejeon, Republic of Korea

**Keywords:** Necroptosis, Cell death and immune response

## Abstract

RIPK3–ZBP1–MLKL-mediated necroptosis is a proinflammatory cell death process that is crucial for antiviral host defence. RIPK3 self-oligomerization and autophosphorylation are prerequisites for executing necroptosis, yet the underlying mechanism of virus-induced RIPK3 activation remains elusive. Interferon-inducible 2′-5′ oligoadenylate synthetase-like (OASL) protein is devoid of enzymatic function but displays potent antiviral activity. Here we describe a role of OASL as a virus-induced necroptosis promoter that scaffolds the RIPK3–ZBP1 non-canonical necrosome via liquid-like phase condensation. This liquid-like platform of OASL recruits RIPK3 and ZBP1 via protein–protein interactions to provide spatial segregation for RIPK3 nucleation. This process facilitates the amyloid-like fibril formation and activation of RIPK3 and thereby MLKL phosphorylation for necroptosis. Mice deficient in *Oasl1* exhibit severely impaired necroptosis and attenuated inflammation after viral infection, resulting in uncontrolled viral dissemination and lethality. Our study demonstrates an interferon-induced innate response whereby OASL scaffolds RIPK3–ZBP1 assembly via its phase-separated liquid droplets to facilitate necroptosis-mediated antiviral immunity.

## Abstract (web summary)

Lee et al. uncover a previously uncharacterized role of OASL in virus-induced necroptosis. OASL chaperones the assembly of RIPK3 and ZBP1 via liquid-liquid phase separation, which induces RIPK3 and necroptosis activation, thereby modulating inflammation and host defence against viral infection.

## Main

Necroptosis is a regulated cell death process that contributes to pathogen-mediated host immune defence by inducing inflammation upon cell death. This intricate balance between induction of inflammatory responses and clearance of pathogens via ‘suicidal death’ is a noteworthy contribution to host–pathogen standoff, as it determines the degree of pathogenic invasion and pathogenicity. Necroptosis signalling is mediated through the formation of a multiprotein complex—the so-called necrosome—in which receptor interacting protein serine/threonine kinase 3 (RIPK3) plays a major effector role. In the absence of caspase-8-mediated apoptosis, tumour necrosis factor (TNF) induces the assembly of the canonical necrosome complex composed of two RIPKs: RIPK1 and the effector RIPK3 (refs. 1–4). More recently, necroptosis has been recognized as an antiviral mechanism after virus infections, such as murine cytomegalovirus (MCMV)^5,6^, herpes simplex virus-1 (HSV-1)^7,8^, vaccinia virus (VACV)^2^ and influenza A virus (IAV)^9^, by forming a non-canonical necrosome composed of RIPK3 and an interferon-stimulated gene (ISG), Z-DNA-binding protein 1 (ZBP1)^10,11^. RIPK3-mediated phosphorylation of its substrate, mixed lineage kinase domain-like pseudokinase (MLKL), triggers executional signals that increase membrane permeabilization for the release of intracellular damage-associated molecular patterns and thereby elicits a robust antiviral immune response^12–17^.

RIPK3 autophosphorylation is the primary prerequisite for its self-activation to relay downstream signals for inducing necroptotic cell death; however, this fundamental mechanism remains hypothetical. It has been proposed that intermolecular interactions mediated by the RIP homotypic interaction motif (RHIM) result in high-order hetero-amyloid structures, which may serve as a platform for RIPK3 autophosphorylation^18–21^. Recent findings have also revealed that artificially induced RIPK3 homo-oligomerization is sufficient to induce necroptosis^22–24^, which suggests that the kinase activity of RIPK3 could be activated by proximity within RIPK3 oligomers. In addition, RIPK3 assembles into discrete functional amyloid-like foci in the cytosol, but it remains unclear how RIPK3 amyloid formation contributes to necroptosis signalling^18,25,26^.

The indispensable role of ZBP1 in virus-induced necroptosis, along with other growing evidence, suggests that robust activation of necroptosis during virus infection requires synergistic interplay between type I interferon (IFN) and TNF signalling to intensify the activation of RIPK3 (refs. 27, 28). During virus infection, rapid activation of type I IFN signalling gives rise to strong expression of ISGs, among which include the oligoadenylate synthetase (OAS) gene family. OAS family proteins belong to the nucleotidyltransferase superfamily and confer protection against viruses through their 2′-5′-phosphodiester-linked oligoadenylates (2-5As) synthetase activity that is triggered after binding to viral double-stranded RNA (dsRNA). Unlike OAS proteins, the protein OAS-like (OASL) lacks enzymatic activity, yet still has regulatory functions in innate immunity, suggesting that OASL has a role in a RNase L-independent antiviral mechanism. Previous studies have shown that OASL has potent target-specific antiviral activity against a panel of RNA and DNA viruses, enhancing antiviral activity in concert with other ISGs^29,30^. By contrast, other studies^31,32^ have reported that binding of OASL to RIG-I enhances IFN production during RNA virus infection, whereas binding to cGAS suppresses IFN production during DNA virus infection. Although the definitive antiviral role of OASL remains unclear, the subcellular localization of OASL appeared as distinct foci in the cytoplasm and the nucleus, which suggests that OASL has a behaviour-specific function. These contentious findings prompt further investigation of the precise role of OASL during virus infections.

Liquid–liquid phase separation (LLPS) is an emerging paradigm in the formation of membraneless biomolecular condensates that enables spatiotemporal regulation of biochemical signalling^33–36^. Liquid droplets serve as a molecular platform to nucleate biomolecules, including proteins and nucleic acids, which discretely tune condition-specific reactions within the complex. Although these liquid condensates are initially highly mobile and dynamic owing to the lack of physical barriers, many proteins, such as FUS, α-synuclein, and STING, undergo phase transition over time to a more rigid hydrogel or amyloid-like fibril structure^35–39^. Thus, phase separation facilitates efficient formation of signalling complexes by inducing concentrated homotypic and heterotypic interactions within the partitioned core.

In this study, we identify OASL as a regulator of virus-induced necroptosis that promotes antiviral activity to restrict viral replication and dissemination. OASL undergoes LLPS and chaperones the assembly of RIPK3–ZBP1 necrosome by recruiting RIPK3 and ZBP1 into its phase-separated droplets via protein–protein interactions. These phase condensates serve as a platform for RIPK3 to nucleate, ultimately inducing RIPK3 amyloid-like fibre formation and enzymatic activation. Subsequent activation of the non-canonical necroptosis pathway elicits antiviral activity by restricting virus replication and dissemination in vivo. Herein, we elucidate the molecular action of the IFN-inducible OASL as a key signalling adaptor for the RIPK3–ZBP1 necroptotic pathway after virus invasion.

## Results

### Identification of OASL as a binding partner of RIPK3

**Fig. 1:**
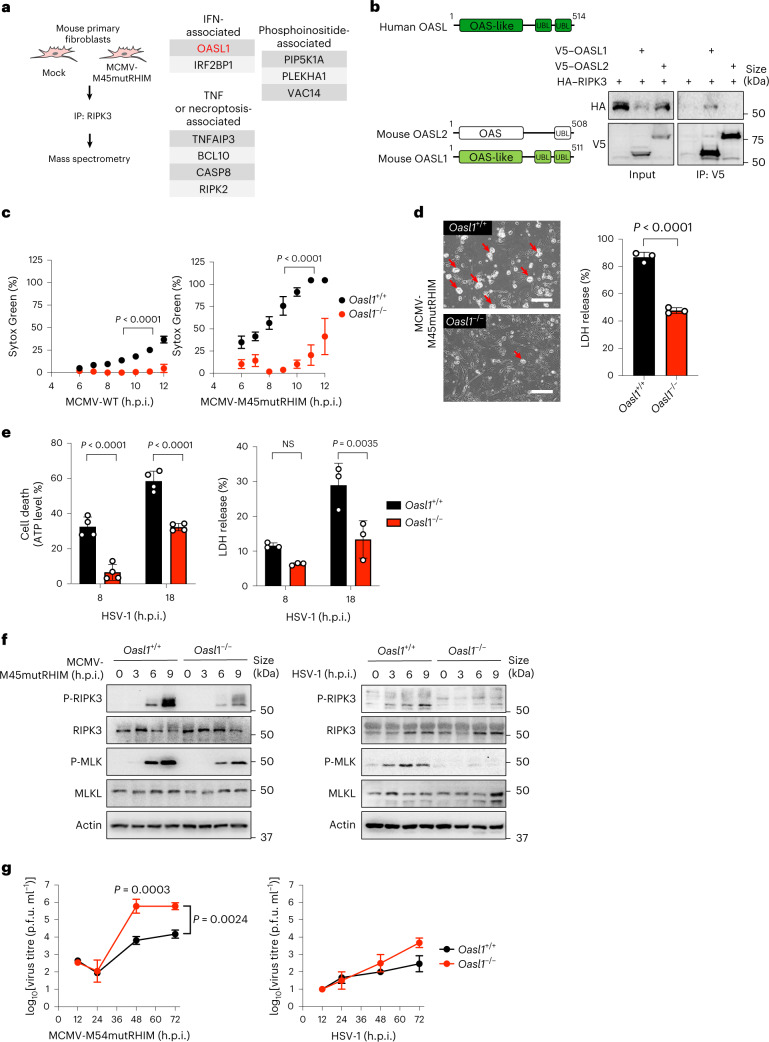
The IFN-stimulated protein OASL is required for efficient virus-induced necroptosis. **a**, Mouse primary fibroblasts were mock-infected or infected with MCMV-M45mutRHIM virus (multiplicity of infection (m.o.i.) = 5) for 6 h. RIPK3 protein complexes were enriched and immunoprecipitated (IP) using RIPK3 antibody-conjugated agarose beads and analysed by mass spectrometry. Functional-related or biological-related proteins are grouped in boxes. **b**, HEK 293T cells were transfected with the indicated constructs, and cell lysates were immunoprecipitated with V5-specific antibody. Immunoprecipitates and whole cell extracts (input) were analysed by immunoblotting with the indicated antibodies. **c**, Cell death kinetics of *Oasl1*^+/+^ and *Oasl1*^*–/–*^ primary fibroblasts infected with MCMV-WT or MCMV-M45mutRHIM (m.o.i. = 5). Necrotic cell death was measured on the basis of the uptake of Sytox Green and quantified in real-time from 4 to 12 h.p.i. (*n* = 4 biological replicates). **d**, Left: microscopy analysis of cell death in *Oasl1*^+/+^ and *Oasl1*^*–/–*^ primary fibroblasts infected with MCMV-M45mutRHIM at 16 h.p.i. Arrows indicate cells with necrotic features after infection. Scale bar, 20 μm. Right: quantification of necrotic cell death by measuring the release of LDH from *Oasl1*^+/+^ and *Oasl1*^*–/–*^ primary fibroblasts infected with MCMV-M45mutRHIM (m.o.i. = 5) for 8 h. **e**, Quantification of necrotic cell death by measuring intracellular ATP levels (*n* = 4) and LDH release (*n* = 3) in supernatants of *Oasl1*^+/+^ and *Oasl1*^*–/–*^ primary fibroblasts infected with HSV-1 (m.o.i. = 5). **f**, Immunoblot analysis of RIPK3 and MLKL phosphorylation (P-RIPK3 and P-MLKL, respectively) in *Oasl1*^+/+^ and *Oasl1*^*–/–*^ primary fibroblasts infected with MCMV-M45mutRHIM (left) or HSV-1 (right) at m.o.i. = 5 for the indicated hours. Infected cells were collected and subjected to immunoblotting with the indicated antibodies. **g**, Viral replication of MCMV-M45mutRHIM and HSV-1 in *Oasl1*^+/+^ (*n* = 4 for MCMV-M45mutRHIM, *n* = 3 for HSV-1) and *Oasl1*^*–/–*^ (*n* = 2) primary fibroblasts were determined by plaque assays by titrating culture supernatants at the indicating time points. Data are representative of two (**b**,**f**,**g**) or three (**c**–**e**) independent experiments. For **c**–**e**,**g**, data are presented as the mean ± s.e.m. Statistical analyses were performed using two-tailed unpaired *t*-test (**d**) or two-way analysis of variance (ANOVA) (**c**–**e**,**g**). NS, not significant. Source data

MCMV-encoded M45 is a viral inhibitor of RIP activation that blocks necroptosis through the disassembly of the RIPK3–ZBP1 non-canonical necrosome. Thus, we utilized a necroptosis-sensitive mutant MCMV carrying the RHIM mutation of M45 (MCMV-M45mutRHIM) to identify key regulators of the necrosome complex in the context of virus infection^5,6^. We infected mouse primary tail fibroblasts with the necroptosis-sensitive MCMV-M45mutRHIM, and at 6 h post-infection (h.p.i.), endogenous mouse RIPK3 was immune-purified and subjected to mass spectrometry analysis. In agreement with previous reports^14^, a number of TNF signalling proteins and phosphoinositide-related enzymes were specifically detected in the RIPK3 complex after virus infection (Fig. 1a and Supplementary Table 1). Notably, an IFN-inducible protein, OASL1, was detected from the RIPK3 complex following MCMV-M45mutRHIM infection. Mouse OASL1 shares high sequence similarity (74%) with human OASL and is predicted to contain an amino-terminal enzymatically inactive OAS domain (N-OAS) and two carboxy-terminal ubiquitin-like (UBL) domains (C-UBL) like human OASL. By contrast, mouse OASL2 preserves the 2-5As synthesis activity within its N-terminal OAS domain and carries a C-terminal single UBL domain. Co-immunoprecipitation assays showed that RIPK3 specifically interacted with OASL1 but not with OASL2 (Fig. 1b).

### OASL is required for efficient virus-induced necroptosis

**Extended Data Fig. 1:**
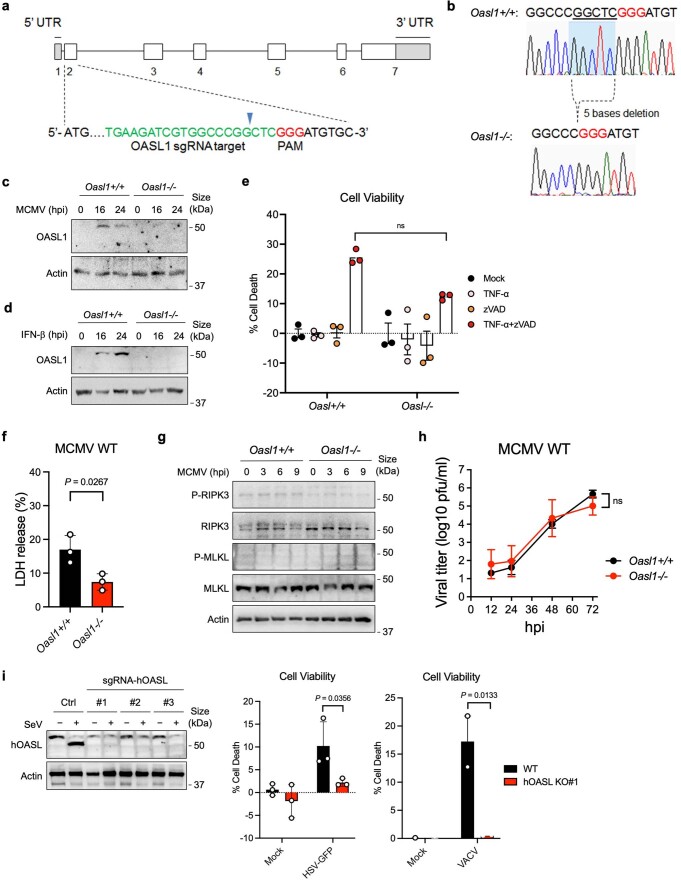
Generation of *Oasl1*-/- mouse using the CRISPR-Cas9 system and the function of *Oasl1* in virus-induced necroptosis. **a**, Schematic of the genomic target in *OASL1* gene and *OASL1* exon 2 sgRNA. The sgRNA sequence (designed in http://chopchop.cbu.uib.no) is marked in green and the protospacer adjacent motif (PAM) sequence is marked in red. Blue arrow points the predicted cleavage site. **b**, Mouse *Oasl1* genomic sequences from *Oasl1*^+/+^ and *Oasl1*^*-/-*^ littermate mice. PAM sequence is marked in red. 5 base pairs deletion was shown in *Oasl1-/-* mice. **c,d**, Immunoblot analysis of OASL1 expression in *Oasl1*^*+/+*^ and *Oasl1*^*-/-*^ primary fibroblasts infected with MCMV (**c**) or treated with IFN-β (**d**) for the indicated times. **e**, Quantification of necrotic cell death by measuring the intracellular ATP levels in *Oasl1*^+/+^ and *Oasl1*^*-/-*^ primary fibroblasts upon TNF-α (50 ng/ml) and/or zVAD (20 mM) treatment. **f**, Measurement of lactate dehydrogenase (LDH) release to the medium in *Oasl1*^*+/+*^ or *Oasl1*^*-/-*^ primary fibroblasts infected with MCMV-WT (m.o.i. = 5) for 8 h. **g**, Immunoblot analysis of RIPK3 and MLKL phosphorylation in *Oasl1*^*+/+*^ or *Oasl1*^*-/-*^ primary fibroblasts infected with MCMV-WT (m.o.i. = 5) for the indicated hours. **h**, MCMV-WT titer in *Oasl1*^+/+^ and *Oasl1*^*-/-*^ primary fibroblasts were determined by plaque assays by titrating culture medium at the indicating time points (*n* = 4 for *Oasl1*^+/+^, *n* = 2 for *Oasl1*^*-/-*^). **i**, (Left) Immunoblot analysis of CRISPR Cas9-mediated knockout of human OASL in A549 cells upon mock or Sendai virus (SeV) infection (Asterisk, non-specific band). Quantification of necrotic cell death by measuring the intracellular ATP levels in WT and hOASL KO A549 cells infected with (middle) HSV-GFP (m.o.i. = 10) upon IFN-β stimulation (*n* = 3) or (right) VACV (m.o.i. = 5) (*n* = 2). **c**,**d**,**g**, Data are representative of two independent experiments. **e**,**f**, Data are presented as mean ± SEM from three independent experiments. Statistical analyses were performed using a two-tailed unpaired *t*-test (**f**) or two-way analysis of variance (ANOVA) (**e**,**h**,**i**). ns, not significant. Source data

To delineate the mechanism of RIPK3–OASL interactions, we used the CRISPR–Cas9 system to generate an OASL1 homozygous null knockout (*Oasl1*^*–/–*^) mouse model (Extended Data Fig. 1a,b). *Oasl1* knockout was confirmed by sequencing and checking the protein expression level in primary tail fibroblasts isolated from wild-type (*Oasl1*^+*/*+^) mice and *Oasl1*^*–/–*^ mice (Extended Data Fig. 1c,d). OASL did not exhibit a crucial role in TNF-mediated canonical necroptosis (Extended Data Fig. 1e), whereas *Oasl1*^+*/*+^ and *Oasl1*^*–/–*^ primary mouse fibroblasts exhibited pronounced differences in virus-induced necroptosis upon infection with wild-type MCMV (MCMV-WT) or MCMV-M45mutRHIM during a real-time cytotoxicity assay (Fig. 1c). *Oasl1*^+*/*+^ fibroblasts showed low levels of cell death upon MCMV-WT infection but near complete death by 11 h.p.i. upon MCMV-M45mutRHIM infection (Fig. 1c). Conversely, both the magnitude and kinetics of cell death were considerably reduced in *Oasl1*^*–/–*^ fibroblasts upon MCMV-M45mutRHIM infection (Fig. 1c, right). MCMV-M45mutRHIM-induced necrotic cell death, featured by cytoplasmic swelling (Fig. 1d, left), was extensive in *Oasl1*^+/+^ fibroblasts starting from 6 h.p.i. and continuously increased until 11 h.p.i., whereas it was weak in *Oasl1*^*–/–*^ fibroblasts (Fig. 1c, right). The level of lactate dehydrogenase (LDH) release upon MCMV-M45mutRHIM infection was significantly lower in *Oasl1*^*–/–*^ fibroblasts than in *Oasl1*^+*/*+^ fibroblasts, but minimal in both cells upon MCMV-WT infection (Fig. 1d, right, and Extended Data Fig. 1f). In addition, HSV-1 infection, which has previously been reported to promote ZBP1–RIPK3-dependent necroptosis^7,8^, led to significantly reduced cell death in *Oasl1*^*–/–*^ fibroblasts compared with *Oasl1*^+/+^ fibroblasts as measured by the levels of LDH release and intracellular ATP (Fig. 1e). These results indicate that OASL1 plays a crucial role in mediating virus-induced necroptotic cell death.

The hallmarks of necroptosis activation are RIPK3 autophosphorylation (human RIPK3 at Ser227; mouse RIPK3 at Thr231 and Ser232)^2,3,40^ and MLKL phosphorylation (human MLKL at Thr357 and Ser385; mouse MLKL at Ser345)^25^. Corresponding to the magnitudes of cell death observed in *Oasl1*^+/+^ and *Oasl1*^*–/–*^ fibroblasts (Fig. 1c), MCMV-M45mutRHIM infection-induced RIPK3 phosphorylation was drastically reduced in *Oasl1*^*–/–*^ fibroblasts compared with *Oasl1*^+/+^ fibroblasts (Fig. 1f, left). Consistently, *Oasl1*^*–/–*^ fibroblasts showed markedly reduced MLKL phosphorylation compared with *Oasl1*^+/+^ fibroblasts (Fig. 1f, left). These correlative patterns of cell death and necroptosis activation were readily observed following necroptosis-sensitive HSV-1 infection, but not after necroptosis-resistant MCMV-WT infection (Fig. 1f, right, and Extended Data Fig. 1g). Consequently, *Oasl1*^*–/–*^ fibroblasts that showed substantially lower levels of necroptosis displayed higher MCMV-M45mutRHIM or HSV-1 titres than *Oasl1*^+/+^ fibroblasts (Fig. 1g). By contrast, MCMV-WT replicated at comparable levels in both *Oasl1*^+/+^ and *Oasl1*^*–/–*^ fibroblasts (Extended Data Fig. 1h). Additionally, CRISPR–Cas9-mediated ablation of *Oasl* in necroptosis-sensitive A549 human lung epithelial cells exhibited a significant reduction in cell death upon HSV tagged with green fluorescent protein (HSV–GFP) or VACV infection (Extended Data Fig. 1i). These data collectively indicate that OASL1 promotes RIPK3–MLKL-mediated necroptosis during virus infection to restrict viral replication.

### OASL chaperones the assembly of the RIPK3–ZBP1 necrosome

**Fig. 2:**
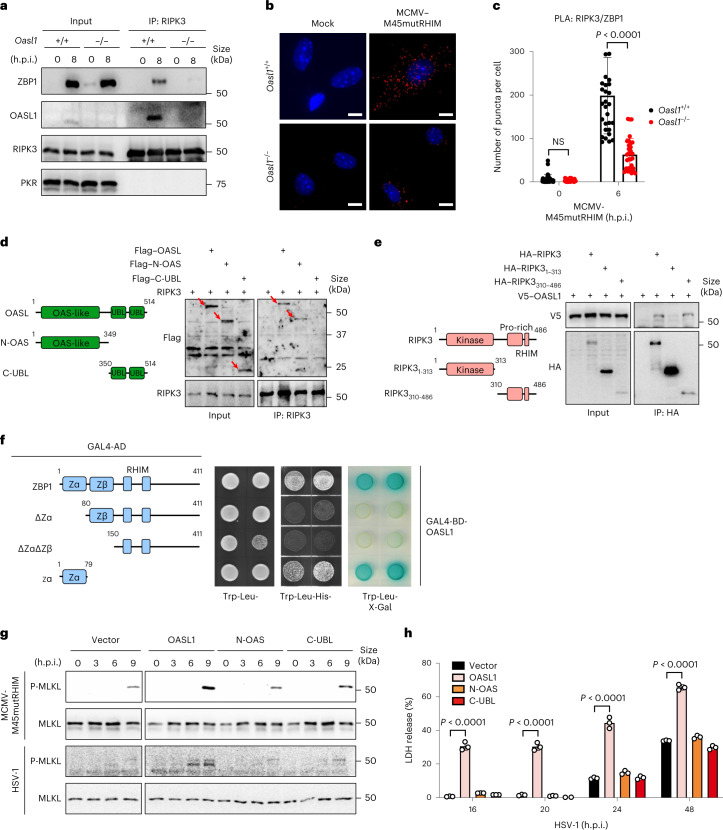
OASL chaperones the assembly of the RIPK3–ZBP1 necrosome complex. **a**, *Oasl1*^+/+^ and *Oasl1*^*–/–*^ primary fibroblasts were infected with MCMV-M45mutRHIM (m.o.i. = 5) for 0 or 8 h. Whole-cell lysates were immunoprecipitated using an anti-RIPK3 antibody, followed by immunoblot analysis using the indicated anti-ZBP1, anti-OASL1, anti-RIPK3 or anti-PKR antibody. **b**,**c**, In situ PLA using anti-RIPK3 and anti-ZBP1 antibodies in *Oasl1*^+/+^ and *Oasl1*^*–/–*^ primary fibroblasts infected with MCMV-M45mutRHIM (m.o.i. = 5) for 0 or 6 h. Fluorescence microscopy was used to detect the discrete fluorescent PLA signals (red dots). Representative images of dots indicate the interaction of endogenous RIPK3 and ZBP1. Scale bar, 10 μm. **c**, Quantification of the dot signals of **b** per cell (*n* = 32 cells). Each dot indicates a single cell. **d**,**e**, HEK 293T cells were transfected with the indicated Flag, HA, V5 or untagged constructs, and cell lysates were immunoprecipitated with anti-RIPK3 (**d**) or anti-HA antibodies (**e**). Immunoprecipitates and whole cell extracts (input) were analysed by immunoblotting with the indicated antibodies. **f**, AH109 yeasts were transformed with pGBKT7-OASL1 or pACT2-ZBP1 constructs. Interactions were determined by triple dropout (Trp-Leu-His-) synthetic medium or secreted α-galactosidase activity assay using X-Gal. **g**, OASL1-mediated phosphorylation of the necroptosis effector MLKL. *Oasl1*^*–/–*^ primary fibroblasts were complemented with empty vector (Vector), HA-tagged full-length OASL1, N-OAS domain or C-UBL domain and infected with MCMV-M45mutRHIM or HSV-1 (m.o.i. = 5) for the indicated times. Total cell lysates were subjected to immunoblot analysis with the indicated antibodies. **h**, Measurement of necrotic cell death on the basis of released LDH in *Oasl1*^*–/–*^ primary fibroblasts reconstituted with empty vector or the indicated OASL1 constructs infected with HSV-1 (m.o.i. = 5) at various time points. Data are representative of two (**b**–**f**) or three (**a**,**g**,**h**) independent experiments. For **c**,**h**, data are presented as the mean ± s.e.m. and statistical analysis was performed using two-way ANOVA. Source data

**Extended Data Fig. 2:**
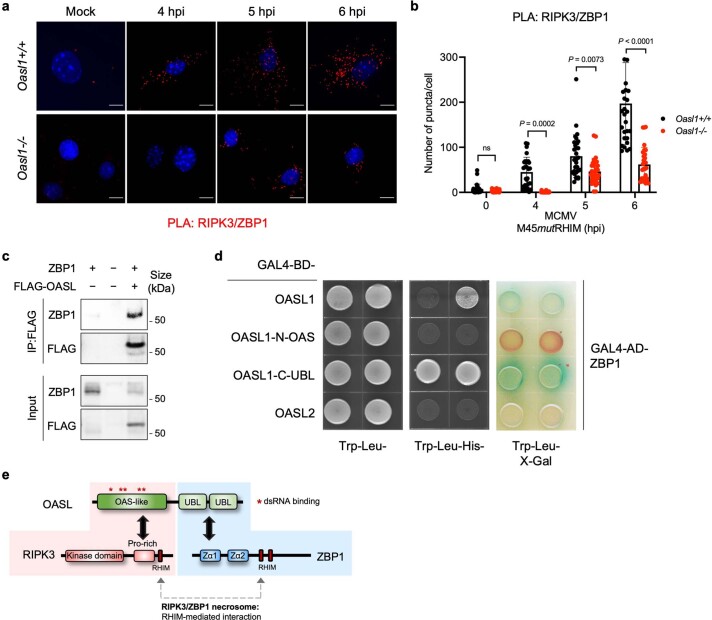
OASL interacts with RIPK3 and ZBP1 through its N-OAS and C-UBL domain, respectively. **a**, *In situ* proximity ligation assay (PLA) of RIPK3 and ZBP1 in *Oasl1*^+/+^ and *Oasl1*^*-/-*^ primary fibroblasts infected with mock or MCMV-M45*mut*RHIM (m.o.i. = 5) for the indicated hours. Fluorescence microscope was used to detect the discrete fluorescent PLA signals (red dots). Representative images of dots indicate the interaction of endogenous RIPK3 and ZBP1. **b**, Quantification of dot signals of **a** per cell (*n* = 30 cells). Each dot indicates single cell. **c**, HEK 293 T cells were transfected with the indicated FLAG or untagged constructs, and cell lysates were immunoprecipitated with anti-FLAG antibody. Immunoprecipitates and whole cell extracts (input) were analyzed by immunoblotting with the indicated antibodies. **d**, AH109 yeasts were transformed with the indicated pGBKT7 or pACT2 constructs. Interactions were measured by triple dropout (Trp-Leu-His-) synthetic medium or secreted α-galactosidase activity assays using X-αGal. **e**, Schematic of the interaction of OASL with RIPK3 and ZBP1 through its N-terminus and C-terminus domains, respectively. **a-d**, Data are representative of two independent experiments with similar results. **b**, Data are presented as mean ± SEM and statistical analysis was performed using two-way ANOVA. Source data

The RHIM-mediated interaction between ZBP1 and RIPK3 forms a non-canonical necrosome during virus infection^6,41,42^. More recently, the Zα domains of ZBP1 were reported to be essential for ZBP1-mediated necroptosis, but the precise role of the Zα domains in RIPK3–ZBP1 interactions during virus infection remains elusive^43,44^. To test whether OASL might play a role in the assembly of the RIPK3–ZBP1 necrosome, MCMV-M45mutRHIM-infected *Oasl1*^+/+^ and *Oasl1*^*–/–*^ primary fibroblasts were collected at 8 h.p.i. for immunoprecipitation with an anti-RIPK3 antibody. RIPK3–ZBP1 interaction was readily detected in *Oasl1*^+/+^ fibroblasts, whereas the interaction was barely detected in *Oasl1*^*–/–*^ fibroblasts (Fig. 2a). In addition, the RIPK3–OASL1 interaction was evidently detected in *Oasl1*^+/+^ fibroblasts (Fig. 2a). Immunoblotting with an anti-PKR antibody was included as a negative control. The RIPK3–ZBP1 interaction was further probed in *Oasl1*^+/+^ and *Oasl1*^*–/–*^ primary fibroblasts by in situ proximity ligation assay (PLA). *Oasl1*^+/+^ primary fibroblasts displayed numerous PLA puncta as early as 4 h.p.i. and gradually increased to a peak at 6 h.p.i., whereas *Oasl1*^*–/–*^ primary fibroblasts exhibited minimal numbers of PLA puncta during the course of infection (Fig. 2b,c and Extended Data Fig. 2a,b). These data suggest that OASL is a crucial mediator in the formation of the RIPK3–ZBP1 necrosome during virus-induced necroptosis.

To further characterize the interaction of OASL and RIPK3, we truncated OASL into the N-OAS domain and the C-UBL domain and identified that RIPK3 bound to N-OAS (Fig. 2d). The serine/threonine kinase RIPK3 has an N-terminal kinase domain and a C-terminal proline-rich region adjacent to the single RHIM (Fig. 2e). Co-immunoprecipitation revealed that the C-terminal proline-rich/RHIM-containing domain, but not the N-terminal kinase domain, of RIPK3 interacted with OASL1 as efficiently as full-length RIPK3 (Fig. 2e). Notably, human OASL also effectively interacted with human ZBP1 in a transient expression assay (Extended Data Fig. 2c). ZBP1 carries two Z-DNA-binding domains (Zα and Zβ) at the N terminus and a pair of adjacent RHIM motifs at the C terminus (Fig. 2f). Owing to the small size of the Zα and Zβ domains, we utilized a yeast two-hybrid system to dissect the protein–protein interaction and revealed that the N-terminal Zα domain of ZBP1 bound to OASL1 as effectively as full-length ZBP1 (Fig. 2f). Furthermore, ZBP1 specifically interacted with OASL1, but not OASL2, and the C-UBL domain of OASL1 was crucial for the interaction with ZBP1 (Extended Data Fig. 2d). To test the functional consequence of these interactions, *Oasl1*^*–/–*^ primary fibroblasts were complemented with full-length OASL or with the N-OAS or C-UBL domain of OASL1, followed by MCMV-M45mutRHIM or HSV-1 infection to induce necroptosis. Compared with expression of the control vector, expression of full-length OASL1 readily enhanced RIPK3-mediated MLKL Ser345 phosphorylation in *Oasl1*^*–/–*^ fibroblasts infected with MCMV-M45mutRHIM or HSV-1 (Fig. 2g). Subsequently, full-length OASL1 expression in *Oasl1*^*–/–*^ primary fibroblasts resulted in significantly higher RIPK3-mediated cell death in HSV-1-infected *Oasl1*^*–/–*^ fibroblasts (Fig. 2g,h). However, neither N-OAS nor C-UBL expression alone was able to reach the level of MLKL phosphorylation and necrotic cell death as robustly as full-length OASL1 expression. This result highlights the requirement of full-length OASL to induce maximal level of necroptosis upon virus infection. Overall, these data indicate that the interactions of OASL with RIPK3 and ZBP1 through its N-OAS and C-UBL domains, respectively, are crucial for RIPK3–ZBP1 assembly and virus-induced necroptosis, thereby establishing OASL as a chaperone for the formation of the non-canonical necrosome (Extended Data Fig. 2e).

### OASL undergoes LLPS

**Fig. 3:**
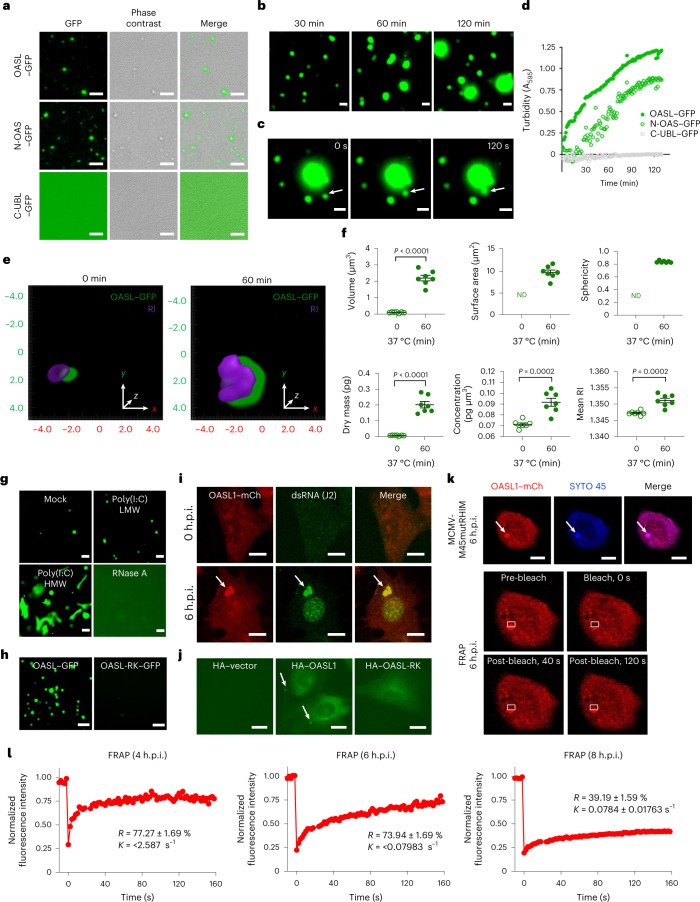
OASL undergoes LLPS during virus-induced necroptosis. **a**, Fluorescence images of droplet formation by GFP-tagged full-length OASL, and the N-OAS and C-UBL domains of OASL in vitro 30 min after induction. **b**, LLPS of GFP-tagged OASL over time in vitro. **c**, Time-lapse images of the coalescence of OASL liquid droplets over 120 s (indicated by the arrow). **d**, Turbidity measurement of GFP-tagged full-length OASL, and the N-OAS and C-UBL domains of OASL over time in vitro. **e**, Representative 3D RI distribution and fluorescence of OASL droplets before and after incubation at physiological conditions (green, GFP fluorescence; purple, RI tomogram; *n* = 50 condensates per condition). **f**, Statistical analyses of morphological (top) and biochemical (bottom) parameters of **e** (*n* = 7 condensates per condition). ND, not detected. **g**, Representative images of OASL–GFP (0.05 μM) phase separation mixed with poly(I:C) LMW (50 μg ml^–1^), poly(I:C) HMW (50 μg ml^–1^) or RNase A (300 μg ml^–1^) for 60 min. **h**, Droplet formation of full-length OASL–GFP and the dsRNA-binding mutant OASL-RK–GFP. **i**, Representative images of *Oasl1*^*–/–*^ primary fibroblasts reconstituted with OASL1–mCherry (OASL1–mCh) and infected with MCMV-M45mutRHIM. Viral dsRNA was immunostained with J2 antibody. **j**, Representative images of *Oasl1*^*–/–*^ primary fibroblasts reconstituted with HA–vector, HA–OASL1 or HA–OASL-RK and infected with MCMV-M45mutRHIM. Arrows indicate discrete OASL1 foci in the cytosol. **k**, Top: arrows indicate the SYTO-45-stained OASL1–mCherry area chosen for photobleaching. Bottom: representative images of OASL1–mCherry foci before and after photobleaching (*n* = 3 cells). Rectangle frames represent the bleached and recovered area within the targeted area. **l**, Quantitative FRAP of OASL1 and SYTO 45 foci for 160 s in *Oasl1*^*–/–*^ fibroblasts reconstituted with OASL1–mCherry and infected with MCMV-M45mutRHIM for 4, 6 or 8 h. Calculated exponential constant (*K*) and normalized plateau after fluorescence recovery (*R*) are the mean ± s.e.m. (*n* = 2 OASL1 and SYTO 45 foci). Images are representative of five (**a**–**c**,**g**,**h**), three (**d**–**f**,**i**,**j**) or two (**k**,**l**) independent experiments. For **f**, data are presented as the mean ± s.e.m. and statistical analysis was performed using two-tailed unpaired *t*-test. Scale bars, 10 μm (**a**–**c**,**g**,**i**–**k**) or 30 μm (**h**). Source data

**Extended Data Fig. 3:**
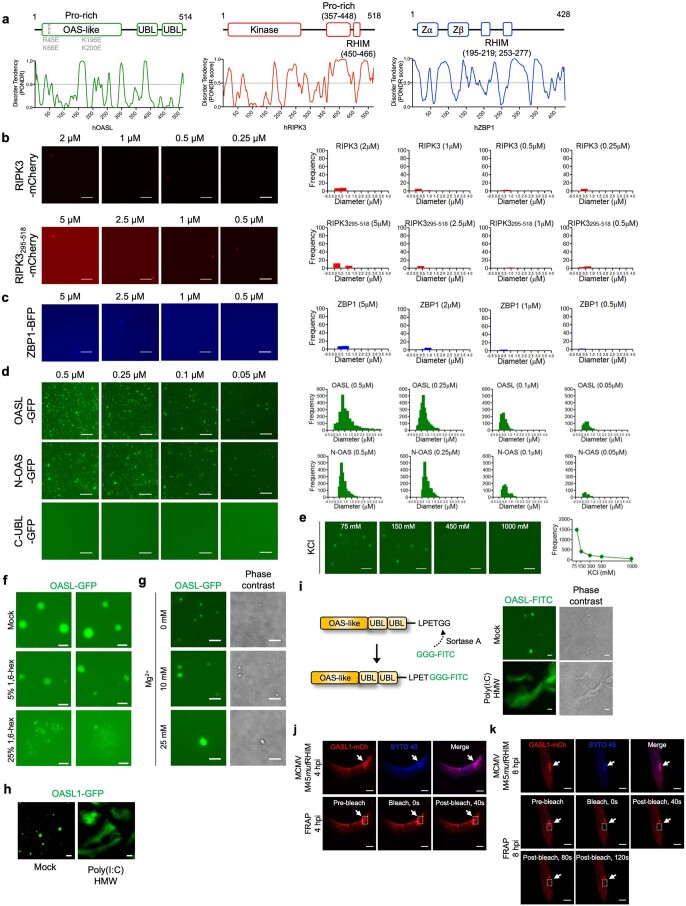
Liquid-liquid phase separation properties of OASL, RIPK3, and ZBP1. **a**, Graph of putative intrinsically disordered regions of human OASL, RIPK3, and ZBP1 as calculated by PONDR (VSL2) algorithm. **b**, Failure of liquid droplet formation of purified mCherry-tagged full-length RIPK3 or C-terminal RIPK3_295-518_. Scale bar, 10 μm. **c**, Failure of liquid droplet formation of purified BFP-tagged full-length ZBP1. Scale bar, 10 μm. **d**, *In vitro* droplet formation by GFP-tagged full-length OASL, N-OAS, or C-UBL protein in a concentration-dependent manner. Incubation was carried out at physiological temperature and buffer for 1 h. Scale bar, 10 μm. **b-d**, Histogram represent distribution of quantified droplet amounts and diameters of the liquid droplets. **e**, Effect of KCl concentration on the formation of OASL liquid droplets. Scale bar, 10 μm. **f**, OASL-GFP droplets treated with increasing concentration of 1,6-hexanediol. Scale bar, 5 μm. **g**, Increase of Mg^2+^ concentration leads to enlargement of OASL droplets. Scale bar, 10 μm. **h**, *In vitro* liquid droplet formation of GFP-tagged mouse OASL (OASL1) treated with mock or poly(I:C) HMW (50 μg/ml) for 1 h. Scale bar, 10 μm. **i**, (Left) Schematic diagram of Sortase A-mediated labeling of FITC at the C-terminus of OASL with Sortase A-recognition motif (LPETGG). (Right) *In vitro* phase separation of FITC-labeled OASL treated with mock or poly(I:C) HMW (50 μg/ml). Scale bar, 10 μm. **j**,**k**, Representative FRAP images of OASL1 foci observed at (**j**) 4 h.p.i. and (**l**) 8 h.p.i. (**l**). SYTO 45-stained OASL1-Cherry foci were chosen for photobleaching. White rectangle box indicates the photobleached and recovered area within the foci. Scale bar, 10 μm. Data are representative of three (**b-i**) or two (**j,k**) independent experiments with similar results. Source data

LLPS drives the formation of membraneless biomolecular condensates, which depends on the presence of intrinsically disordered regions within the proteins^45^. Notably, a computational algorithm-based predictor, PONDR^46^, revealed multiple intrinsically disordered regions distributed throughout the sequences of human OASL, RIPK3 and ZBP1 (Extended Data Fig. 3a). To assess the potential phase separation capability of these proteins, full-length and fragments of OASL fused with enhanced GFP (eGFP), RIPK3–mCherry and ZBP1 tagged with blue fluorescent protein (ZBP1–BFP) were purified from bacteria and subjected to phase separation in vitro. Neither RIPK3 (full-length and RIPK3_295–518_) nor ZBP1 was able to undergo phase separation (Extended Data Fig. 3b,c). By contrast, OASL formed liquid droplets under physiological pH, salt concentration and temperature at a minimum concentration of 0.05 μM (Fig. 3a). The frequency and size of the OASL droplets correlatively increased with the increasing concentrations of OASL (Extended Data Fig. 3d). Notably, N-OAS was sufficient to undergo LLPS as efficiently as full-length OASL, whereas C-UBL did not form any liquid droplets (Fig. 3a and Extended Data Fig. 3d). The dynamic activity of OASL droplets was demonstrated by the increase in droplet size over time (Fig. 3b), whereby liquid droplets coalesced after coming into contact with one another to form larger droplets (Fig. 3c). Furthermore, a time-course spectrometric assay that measures protein turbidity showed a considerable increase in OASL or N-OAS phase separation, but not for C-UBL (Fig. 3d). In accordance with previous studies^47,48^, increasing the salt concentration or incubation with the aliphatic chemical 1,6-hexanediol considerably disrupted OASL droplet formation (Extended Data Fig. 3e,f). Conversely, as divalent cations control phase separation^49,50^, increasing the concentration of Mg^2+^ ions increased the size of OASL droplets (Extended Data Fig. 3g).

Refractive index (RI), a measure of intrinsic optical property, enables the characterization of intracellular structures in a label-free and noninvasive manner^51,52^. A combination of three-dimensional (3D) holotomographic and fluorescence imaging of purified OASL–GFP liquid droplets defined OASL droplets at specific RI values, which enabled quantitative measurement of the morphological and biochemical properties of OASL droplets (Fig. 3e,f). 3D RI tomograms quantitatively demonstrated the increases in volume and surface area of the OASL droplets, as well as the mass and concentration of OASL within the droplets after phase-separation induction. The results correlated with the increased mean RI values (Fig. 3f), which indicated that the droplets fuse over time. In addition, the average sphericity index of the OASL droplets was 0.8368 ± 0.005769 after phase-separation induction, which indicated that the droplets are a near-perfect spherical shape (Fig. 3f). These results suggest that OASL, but not RIPK3 or ZBP1, forms liquid droplets that are fused over time and increase in size and mass.

N-OAS that sufficiently undergoes phase separation contains a dsRNA-binding groove^53^. Notably, OASL droplet formation increased in frequency following treatment with poly(I:C) low molecular weight (LMW), and a large increase in frequency and size was observed after longer treatment with poly(I:C) high molecular weight (HMW) in vitro (Fig. 3g). As seen with OASL–GFP, fluorescence-labelled untagged human OASL, as well as mouse OASL1–GFP, formed phase-separated droplets that were further enhanced after poly(I:C) HMW treatment (Extended Data Fig. 3h–i). Accordingly, RNase A treatment disrupted in vitro OASL phase separation (Fig. 3g). Furthermore, a dsRNA-binding-deficient OASL mutant (OASL-RK; R45E, K66E, R196E and K201E) failed to undergo LLPS in vitro (Fig. 3h). These results indicate that binding of dsRNA contributes to the in vitro phase separation of OASL.

### OASL phase condensates after virus infection

To assess the biological role of OASL phase separation in the context of virus infection, *Oasl1*^*–/–*^ primary fibroblasts expressing WT OASL1–mCherry or the dsRNA-binding mutant OASL1-RK–mCherry were infected with MCMV-M45mutRHIM. WT OASL1–mCherry was observed as discrete cytosolic foci after MCMV-M45mutRHIM infection that specifically colocalized with dsRNA-containing granules stained by the dsRNA-specific antibody J2 (ref. 54) (Fig. 3i). By contrast, the OASL1-RK mutant showed little or no cytoplasmic foci formation upon MCMV-M45mutRHIM infection (Fig. 3j). We next examined the dynamics of OASL1 foci in virus-infected live cells using fluorescence recovery after photobleaching (FRAP) assay. Cytoplasmic foci stained with SYTO Blue, a cell-permeable double-stranded nucleic acid dye, were extensively colocalized with OASL1–mCherry foci in MCMV-M45mutRHIM-infected *Oasl1*^*–/–*^ primary fibroblasts (Fig. 3k). After partially photobleaching a section of the SYTO-stained OASL1–mCherry foci, the fluorescent signal underwent rapid and near-complete recovery by 120 s after bleaching (Fig. 3k, bottom, and Supplementary Video 1). These OASL droplets featured dynamic liquid-like phase condensation and continuous exchange between the foci and the surrounding environment during virus-induced necroptosis (Supplementary Video 1). To further assess whether the OASL droplets display propensity for liquid-to-solid-like transition, FRAP curves were acquired during early and late time points of MCMV-M45mutRHIM infection. Notably, the fluorescence recovery of the droplets was faster and higher at 4 and 6 h.p.i. than at 8 h.p.i. (Fig. 3l, Extended Data Fig. 3j,k and Supplementary Videos 1–3). As liquid droplets exhibit changes in viscoelasticity during droplet maturation^37^, our data suggest that OASL1 foci may manifest a transition from a liquid-like state to a gel-like state over time during virus infection. Overall, these results demonstrate that OASL dynamically phase condensates into dsRNA-containing liquid droplets during virus-induced necroptosis.

### OASL recruits RIPK3 and ZBP1 into its phase condensate to activate RIPK3

**Fig. 4:**
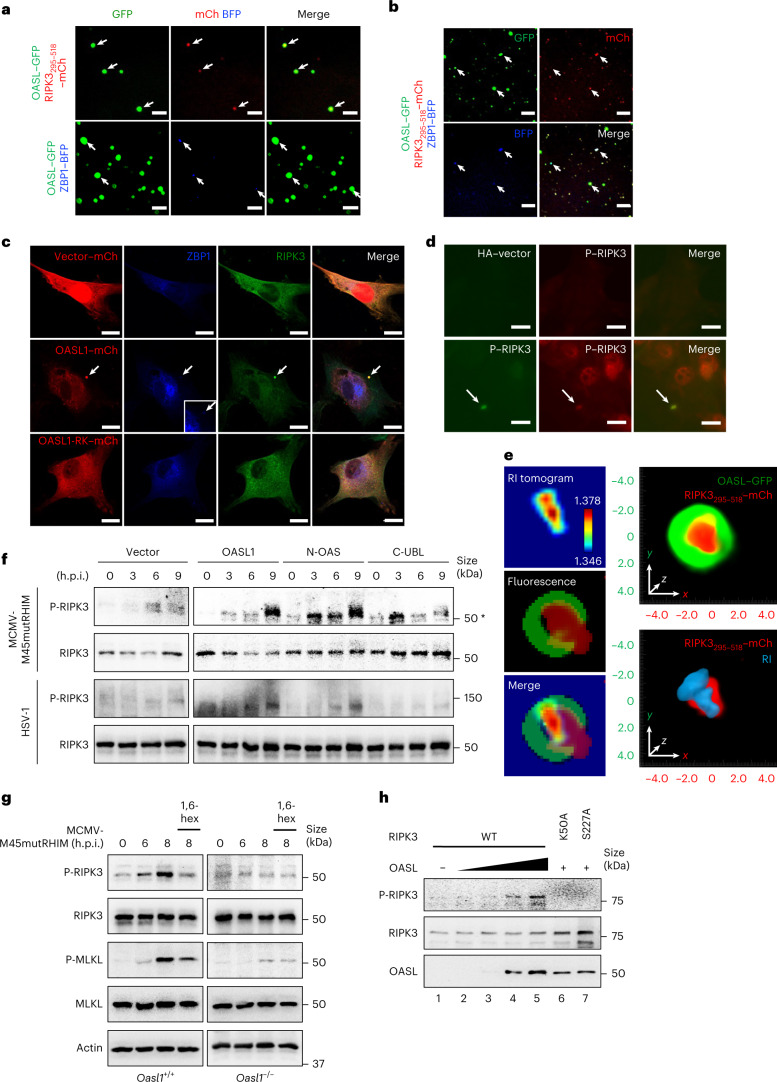
OASL liquid droplets recruit RIPK3 and ZBP1 to activate RIPK3. **a**, Phase separation of OASL–GFP (0.05 μM) incubated with RIPK3_295–518_–mCherry (top; 2 μM) or ZBP1–BFP (bottom; 1 μM). **b**, Representative images of OASL–GFP, RIPK3_295–518_–mCherry and ZBP1–BFP phase separation 1 h after induction. **a**,**b**, Arrows indicate colocalized droplets. **c**, *Oasl1*^*–/–*^ fibroblasts reconstituted with mCherry-tagged vector, OASL1 or OASL1-RK, infected with MCMV-M45mutRHIM and immunostained for endogenous ZBP1 and RIPK3. Arrows indicate complex stained positive for OASL1, ZBP1 and RIPK. Inset represents a zoomed view of cytosolic ZBP1 foci. **d**, MCMV-M45mutRHIM-infected *Oasl1*^*–/–*^ primary fibroblasts reconstituted with empty vector or HA-tagged full-length OASL1. Arrow indicates OASL1 and P-RIPK3 foci in the cytosol. **e**, Left: 2D RI tomogram of RIPK3_295–518_–mCherry and fluorescence image of OASL–GFP and RIPK3_295–518_–mCherry. Right, top: 3D fluorescence image of OASL–GFP and RIPK3_295–518_–mCherry. Right, bottom: 3D fluorescence and RI tomogram of RIPK3_295–518_–mCherry. **f**, Immunoblot of *Oasl1*^*–/–*^ primary fibroblasts complemented with HA-tagged vector, OASL1, N-OAS or C-UBL and infected with MCMV-M45mutRHIM or HSV-1 (m.o.i. = 5) for the indicated times (asterisk indicates a nonspecific band). **g**, Immunoblot of RIPK3 and MLKL phosphorylation in *Oasl1*^+*/*+^ and *Oasl1*^*–/–*^ primary fibroblasts infected with MCMV-M45mutRHIM (m.o.i. = 5) for the indicated hours and pretreated with or without 1% 1,6-hexanediol (1,6-hex) for 2 h. **h**, In vitro kinase reactions were conducted by incubating with purified human WT RIPK3 or a kinase-dead mutant (K50A) or a phosphorylation-deficient mutant (S227A) of human RIPK3 with increasing amounts of purified human OASL protein. Reactions were subjected to SDS–PAGE, and the Ser227 autophosphorylation and input levels of RIPK3 and OASL were evaluated. Data are representative of five (**a**,**b**,**e**) or three (**c**,**d**,**f**,**g**,**h**) independent experiments, with similar results obtained. Scale bars, 10 μm (**a**–**d**). Source data

**Extended Data Fig. 4:**
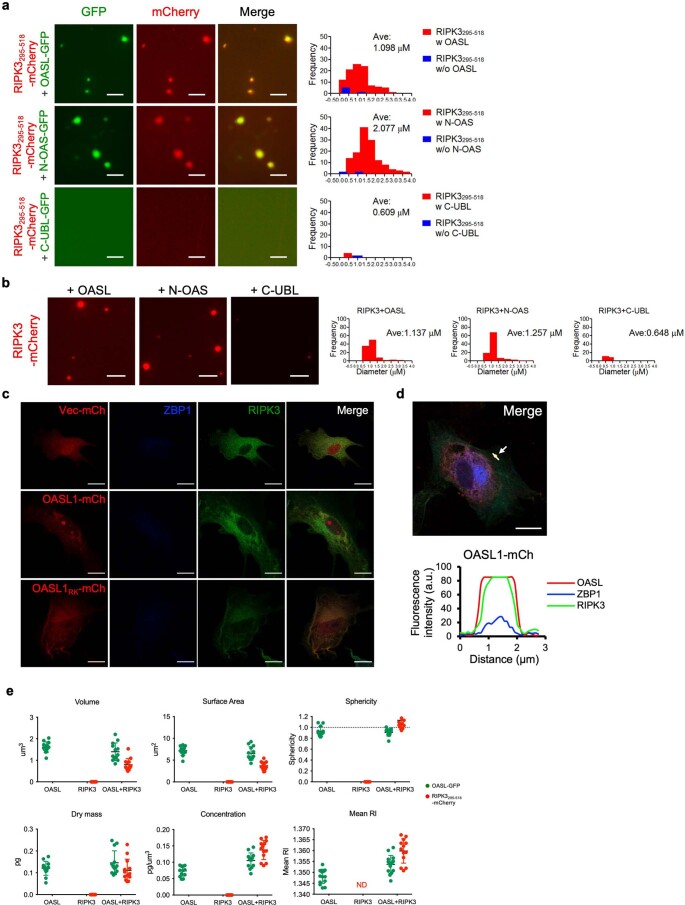
OASL liquid phase separation during virus-induced necroptosis. **a**, (Left) Representative images of liquid droplet formation of GFP-tagged full-length OASL, N-terminus OAS-like domain, or C-terminus UBL domain with mCherry-tagged RIPK3_295-518_. Scale bar, 5 μm. (Right) Histogram of the size and formation frequency of RIPK3_295-518_-mCherry droplets with or without the presence of GFP-tagged OASL proteins. **b**, (Left) Full length RIPK3-mCherry undergoes phase separation in the presence of purified OASL or N-OAS, but not C-UBL. Representative images of liquid droplet formation of mCherry-tagged full-length RIPK3 in the presence of full-length OASL, N-OAS or C-UBL. 2 µM of RIPK3-mCherry was mixed with 0.5 μM of purified OASL proteins. Scale bar, 5 μm. (Right) Histogram of the size and formation frequency of RIPK3 droplets. **c**, Confocal imaging of mock-infected *Oasl1*^*-/-*^ primary fibroblasts reconstituted with mCherry-tagged vector, OASL1, or OASL1_RK_. Scale bar, 10 μm. **d**, (Top) OASL1-mCherry merged image of Fig. 4c with line across the condensate. Scale bar, 10 μm. (Bottom) Line profile of fluorescence intensity indicates colocalization of OASL1, ZBP1, and RIPK3 signal in OASL1-mCherry expressing *Oasl1*^*-/-*^ primary fibroblasts. **e**, Quantitative analysis of morphological (top) and biochemical (bottom) parameters of OASL-GFP alone, RIPK3_295-518_-mCherry alone, and OASL-GFP with RIPK3_295-518_-Cherry liquid-like droplets before and after incubation at physiological conditions (*n* = 13 condensates per group). Morphological parameters include volumes, surface areas, and sphericity. Biochemical parameters include dry mass, concentration, and mean RI. ND, not detected. Data are representative of three (**a-c**) or two (**e**) independent experiments with similar results. Source data

Although purified RIPK3 or ZBP1 was unable to undergo phase separation in vitro (Extended Data Fig. 3b,c), incubation with OASL–GFP efficiently recruited RIPK3_295–518_–mCherry and ZBP1–BFP into OASL droplets in vitro (Fig. 4a). The C-terminal RIPK3_295–518_ that mediated interactions with OASL was sufficient for recruitment into either full-length OASL or N-OAS droplets, but not C-UBL (Extended Data Fig. 4a). mCherry-tagged full-length RIPK3 incubated with purified untagged OASL, N-OAS or C-UBL also showed efficient droplet formation following incubation with OASL or N-OAS, but not C-UBL (Extended Data Fig. 4b). Furthermore, induction of all three proteins together showed that both RIPK3_295–518_–mCherry and ZBP1–BFP were sequestered into OASL–GFP droplets, forming a triple protein condensate in vitro (Fig. 4b). To assess whether RIPK3 and ZBP1 condensed together into OASL liquid droplets during virus infection, *Oasl1*^*–/–*^ primary fibroblasts complemented with OASL1–mCherry were infected with MCMV-M45mutRHIM and subsequently immune-stained for endogenous RIPK3 and ZBP1. Expression of OASL1–mCherry effectively sequestered both RIPK3 and ZBP1 into its condensate in the cytoplasm during virus infection (Fig. 4c and Extended Data Fig. 4c,d). By contrast, RIPK3 and ZBP1 were diffusely localized in MCMV-M45mutRHIM-infected *Oasl1*^*–/–*^ fibroblasts expressing vector or OASL1-RK–mCherry (Fig. 4c).

*Oasl1*^*–/–*^ primary fibroblasts reconstituted with haemagglutinin-tagged OASL1 (HA–OASL1) formed discrete cytosolic condensates after MCMV-M45mutRHIM infection that precisely colocalized with phospho-RIPK3-positive foci. By contrast, no specific staining was observed in vector-expressing cells (Fig. 4d). 3D holotomography fluorescence imaging of purified OASL–GFP and RIPK3_295–518_–mCherry also revealed that RIPK3 was highly concentrated within OASL droplets (Fig. 4e). RI tomograms displayed an increase in RI values towards the core of the OASL droplet, ranging from 1.346 to 1.378, which typically define protein condensates^55^ (Fig. 4e, left). Moreover, the volume, concentration and mean RI of RIPK3_295–518_–mCherry were substantially increased after incubation with OASL–GFP (Extended Data Fig. 4e). To further examine the effect of OASL phase separation on RIPK3 kinase activity, *Oasl1*^*–/–*^ primary fibroblasts were complemented with OASL1, N-OAS or C-UBL, followed by MCMV-M45mutRHIM or HSV-1 infection. Expression of OASL1 or N-OAS induced robust RIPK3 autophosphorylation, whereas expression of C-UBL or vector marginally induced autophosphorylation (Fig. 4f). Furthermore, treatment with 1,6-hexanediol, which disrupts OASL droplet formation (Extended Data Fig. 3f), led to a considerable reduction in RIPK3 and MLKL phosphorylation in MCMV-M45mutRHIM-infected *Oasl1*^+/+^ primary fibroblasts (Fig. 4g). In addition, in vitro kinase reaction assays demonstrated that increasing the amount of OASL correlatively increased RIPK3 autophosphorylation level, whereas no effect was observed on the phosphorylation of a RIPK3-K50A kinase-dead mutant or a RIPK3-S227A phosphorylation-dead mutant (Fig. 4h). Both the in vitro phase-separation assays and the virus infection study collectively show that OASL drives the condensation of RIPK3 and ZBP1 by recruiting them into its liquid droplets via protein–protein interactions, which ultimately induces RIPK3 autophosphorylation.

### OASL phase condensate promotes RIPK3 amyloid-like fibril formation

**Fig. 5:**
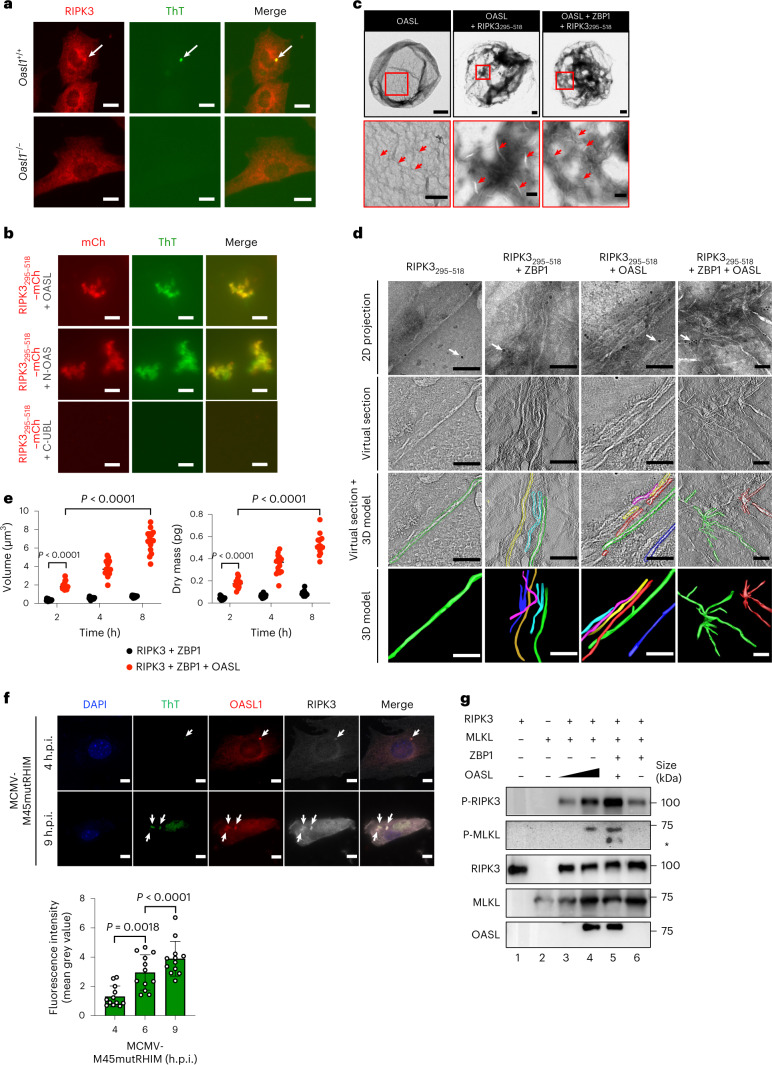
OASL phase condensates nucleate RIPK3 and promote RIPK3 activation and amyloid formation. **a**, *Oasl1*^+/+^ and *Oasl1*^*–/–*^ primary fibroblasts infected with MCMV-M45mutRHIM and immunostained for endogenous RIPK3 (anti-RIPK3) and amyloid-like structure (ThT). Arrow indicates foci positive for RIPK3 and ThT. **b**, Filamentous structure formation after mixing RIPK3_295–518_–mCherry with high concentrations (2.5 μM) of purified OASL, N-OAS or C-UBL. **c**, Top: TEM images of OASL–GFP alone, with RIPK3_295–518_–mCherry or with RIPK3_295–518_–mCherry and ZBP1–BFP after phase-separation induction for 2 h. Bottom: magnified images of the red square area for each image showing fibril-like structures (red arrows) inside the OASL droplet. **d**, Electron tomogram of RIPK3_295–518_–mCherry amyloids alone, with ZBP1–BFP, OASL–GFP, or with ZBP1–BFP and OASL–GFP. First row: 2D projections of the amyloid fibrils. Arrows indicate gold particles used for image alignment. Second row: virtual sections through the tomogram at different *z* axis positions. Third row: overlay of virtual sections and 3D models. Fourth row: reconstructed 3D model of the amyloid fibrils. **e**, Quantitative analysis of 3D tomograms of RIPK3–mCherry after incubation with ZBP1 or with ZBP1 and OASL at the indicated times (*n* = 15). **f**, Top: *Oasl1*^*–/–*^ fibroblasts reconstituted with OASL1–mCherry, infected with MCMV-M45mutRHIM and immunostained for amyloid-like structures. Arrows indicate foci positive for OASL1, RIPK3 and ThT. Bottom: quantification of ThT mean fluorescence intensity per time point (*n* = 12). **g**, In vitro kinase reactions of purified OASL–GFP, GST–RIPK3, ZBP1–BFP and MLKL. OASL–RIPK3–ZBP1 were induced for phase separation, subjected to kinase reaction and immunoblotted for Ser227 autophosphorylation of RIPK3 and Ser358 phosphorylation of MLKL (asterisk indicates a nonspecific band). Data are representative of three (**a**,**b**,**e**–**g**) independent experiments, with similar results obtained. For **c,d**, TEM images are representative of at least eight fields with four independent experiments. For **e**,**f**, data are presented as the mean ± s.e.m. and statistical analyses were performed using two-way (**e**) or one-way (**f**) ANOVA. Scale bars, 100 nm (**d**), 200 nm (**c**, bottom), 500 nm (**c**, top) or 10 μm (**a**,**b,f**). Source data

**Extended Data Fig. 5:**
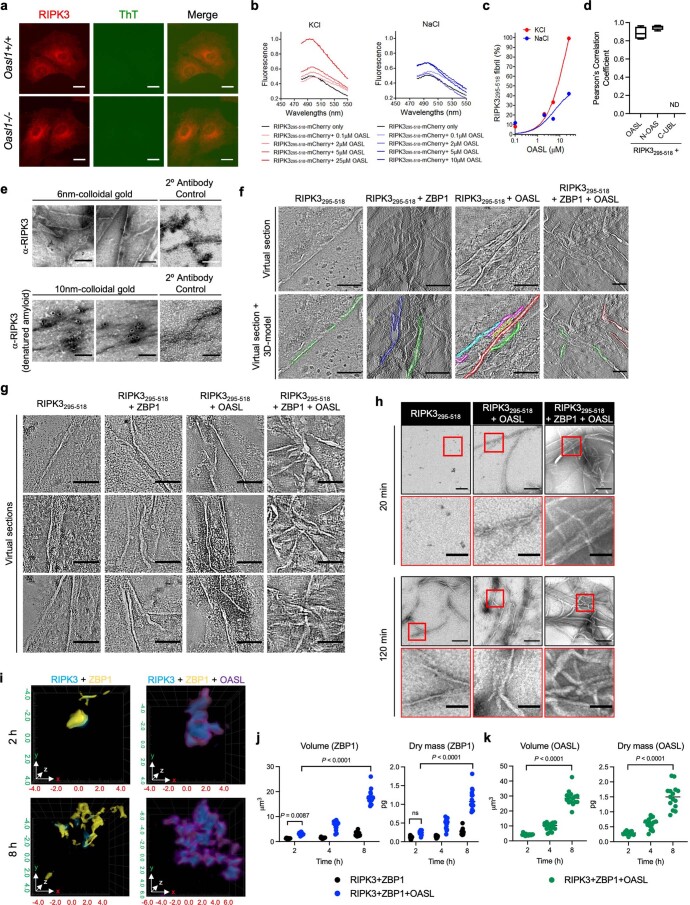
OASL phase separation promotes RIPK3 amyloid fibrillation. **a**, Representative images of mock-infected *Oasl1*^+/+^ and *Oasl1*^*-/-*^ primary fibroblasts immunostained for endogenous RIPK3 and amyloid-like structure by anti-RIPK3 antibody and Thioflavin T (ThT), respectively. Scale bar, 10 μm. **b**, Fibrillation of RIPK3_295-518_-mCherry in vitro with increasing amounts of OASL at physiological (left) KCl or (right) NaCl concentration for 16 h. **c**, Dose-response curve of OASL inducing RIPK3_295-518_-mCherry fibrillation assessed by ThT emission. **d**, Quantification of RIPK3_295-518_-mCherry and ThT signal colocalization upon incubation with OASL, N-OAS, or C-UBL. Box plots show the minimum, first quartile, median, third quartile, and maximum with *n* = 150 droplets per group. **e**, TEM imaging of RIPK3_295-518_-mCherry fibrils after phase separation with ZBP1-BFP and OASL-GFP, followed by immunogold-labeling with anti-RIPK3 antibody upon (top) normal or (bottom) denatured condition. Prominent immunogold labeling was observed in denatured condition. **f**, Virtual sections of RIPK3 amyloid fibrils through the tomogram and overlays with 3D-model at different z-axis positions. **g**, Virtual sections of RIPK3_295-518_-mCherry fibrils through tomogram at different z-axis positions. **h**, TEM images of RIPK3_295-518_-mCherry alone or incubated with OASL or OASL and ZBP1 together after 20 or 120 min of phase separation. Scale bar, 200 nm. Insets: magnified views of the red box regions. **e-h**, Scale bar, 100 nm. **i**, Representative 3D refractive index distribution of RIPK3 + ZBP1 or RIPK3 + ZBP1 + OASL at the indicated times. RI tomogram: blue (RIPK3), yellow (ZBP1), purple (OASL). (*n* = 30 condensates per condition). **j,k**, 3D tomogram quantitative analysis of ZBP1-BFP **(j)** and OASL-GFP **(k)** upon co-incubation with RIPK3 (*n* = 15 condensates per group). Data are representative of three (**a-d**) or two (**i-k**) independent experiments with similar results. **e-h**, TEM images are representative of at least 8 fields with four independent experiments. **j,k**, Data are presented as mean ± SEM and statistical analyses were performed using two-way ANOVA. ND, not detected. Source data

Notably, staining with thioflavin T (ThT), an aromatic cross β-sheet-specific dye for amyloid-like structures, detected endogenous RIPK3 foci in MCMV-M45mutRHIM-infected *Oasl1*^+/+^ primary fibroblasts that resembled previously reported RIPK3 amyloid fibrils (Fig. 5a) (ref. 18). By contrast, MCMV-M45mutRHIM-infected *Oasl1*^*–/–*^ or mock-infected *Oasl11*^+/+^ and *Oasl1*^*–/–*^ primary fibroblasts showed diffuse cytoplasmic localization of endogenous RIPK3 without any distinct ThT-positive staining (Fig. 5a and Extended Data Fig. 5a). Furthermore, RIPK3_295–518_–mCherry fibril formation substantially increased after in vitro incubation with OASL in a dose-dependent manner (Extended Data Fig. 5b,c). In particular, KCl was more efficient in inducing OASL-mediated RIPK3_295–518_–mCherry fibril formation than NaCl (Extended Data Fig. 5c). Finally, ThT-positive RIPK3_295–518_–mCherry fibrils were readily observed after in vitro incubation with OASL or N-OAS, but not with C-UBL (Fig. 5b and Extended Data Fig. 5d). These data collectively indicate that the N-OAS domain of OASL is vital for efficient RIPK3 fibril formation.

Phase-separated structures provide a platform for protein nucleation, which often results in a transition from a highly mobile liquid-state to a gel-like state owing to the increase in viscoelasticity^37,38,56,57^. High-resolution transmission electron microscopy (TEM) showed that OASL formed dense, fibril-like structures inside the liquid droplets (Fig. 5c). Incubation of OASL with RIPK3_295–518_ induced amyloid-like fibril formation within the OASL liquid droplets (Fig. 5c). These RIPK3_295–518_ amyloid fibrils, detected by anti-RIPK3 immunogold labeling, within the OASL droplets were dense and extensively branched following incubation with ZBP1 (Fig. 5c and Extended Data Fig. 5e). 3D electron tomograms reconstructed from consecutive virtual sections of TEM images with incrementally tilted angles revealed that whereas RIPK3_295–518_ alone formed irregular and short fibril intermediates^18^, incubation with ZBP1 or OASL led to elongated amyloid fibril formation (Fig. 5d and Extended Data Fig. 5f,g). In particular, incubation with OASL led to highly ordered and homomorphic amyloid formation, whereas incubation with the RHIM-containing ZBP1 resulted in irregular and polymorphic amyloid structures (Fig. 5d and Extended Data Fig. 5f,g). Finally, RIPK3_295–518_ amyloids were substantially ordered and highly divaricated after incubation with both OASL and ZBP1 (Fig. 5d and Extended Data Fig. 5f,g). Time-dependent and interaction-dependent amyloidogenesis of RIPK3_295–518_ was noted by the growth of RIPK3_295–518_ fibrils after long in vitro incubation (120 min) compared with short incubation (20 min), especially those formed by RIPK3_295–518_ alone (Extended Data Fig. 5h). Holotomography imaging also revealed that RIPK3 increasingly nucleated over time as OASL droplets grew in size and concentration in vitro. This RIPK3 nucleation was not observed after incubation with ZBP1 alone (Fig. 5e and Extended Data Fig. 5i–k), which highlights the role of OASL phase condensation in nucleating RIPK3 and promoting RIPK3 amyloid fibril formation. The growth and maturation of RIPK3 amyloid-like fibrils within the OASL droplets were further evidenced in MCMV-M45mutRHIM-infected primary fibroblasts by the colocalization of ThT-positive OASL1 and RIPK3 foci and the increasing ThT signal over time (Fig. 5f).

To further demonstrate that fibrils of and activated RIPK3 directly transduce downstream signalling to its substrate MLKL, an in vitro kinase reaction was performed after induction of phase separation of OASL, RIPK3 and ZBP1 followed by the addition of MLKL and ATP to induce the kinase reaction. OASL phase separation regulated RIPK3 autophosphorylation, which subsequently induced MLKL phosphorylation (Fig. 5g). However, the RHIM-mediated interaction between RIPK3 and ZBP1 was not sufficient for inducing RIPK3 amyloid fibril formation and MLKL phosphorylation without OASL (Fig. 5d,e,g and Extended Data Fig. 5f–k). These data demonstrate that the recruitment of RIPK3 and ZBP1 into OASL droplets results in pervasive amyloid formation of RIPK3 during virus infection, which indicates that OASL phase condensation is a spatial hub for nucleating RIPK3 to accelerate amyloid fibril formation.

### OASL1-mediated necroptosis restricts MCMV replication and inflammation in vivo

**Fig. 6:**
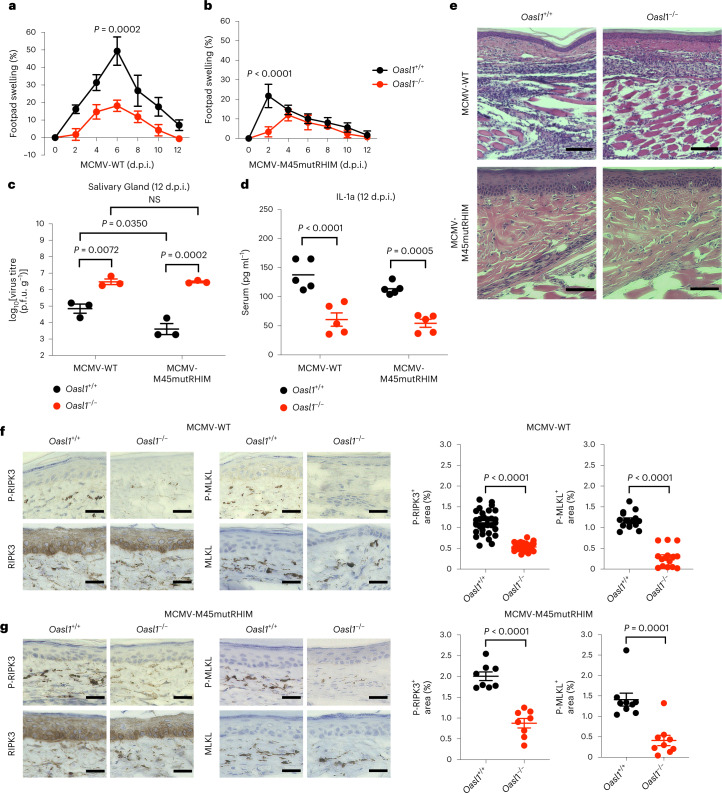
OASL1-mediated necroptosis restricts MCMV viral replication and inflammation in vivo. **a**,**b**, Time course measurement of footpad swelling caused by subcutaneous footpad injection of age-matched and sex-matched *Oasl1*^+/+^ and *Oasl1*^*–/–*^ mice with 10^6^ p.f.u. MCMV-WT (**a**) (*Oasl1*^+/+^, *n* = 8; *Oasl1*^*–/–*^, *n* = 6 for 0–10 d.p.i., *n* = 2 for 12 d.p.i.) or MCMV-M45mutRHIM (**b**) (*Oasl11*^+/+^, *n* = 8 for 0–6 d.p.i., *n* = 4 for 8–12 d.p.i.; *Oasl1*^*–/–*^, *n* = 8, except *n* = 2 for 6 d.p.i.). The thickness of the footpads was measured using a digital caliper. Data are presented as the percent increase in thickness relative to the pre-injection measurement and plotted as mean values at the indicated times (*n* = 9 mice per genotype). **c**, Virus titres in SGs of *Oasl1*^+/+^ and *Oasl1*^*–/–*^ mice at 12 d.p.i. of MCMV-WT or MCMV-M45mutRHIM were determined by plaque assay. log_10_(2) is the limit of detection owing to the toxicity of SG homogenates in NIH3T3 cells (*n* = 3 mice per genotype). **d**, IL-1α levels in the serum of *Oasl1*^+/+^ and *Oasl1*^*–/–*^ mice at 12 d.p.i. of MCMV-WT or MCMV-M45mutRHIM were determined by ELISA (*n* = 5 mice per genotype). **e**, Haematoxylin and eosin staining for histological analysis of footpads from mice infected with MCMV-WT (day 6) or MCMV-M45mutRHIM (day 2). **f**,**g**, Left: footpad sections corresponding to **e** (MCMV-WT (**f**) and MCMV-M45mutRHIM (**g**)) were subjected to immunohistochemistry staining of RIPK3, P-RIPK3, MLKL and P-MLKL. Positive staining appears brown with haematoxylin counterstain. Right: quantification of the areas stained by P-RIPK3 and P-MLKL in mice infected with MCMV-WT (**f**, *n* = 30 for P-RIPK3, *n* = 15 for P-MLKL) or MCMV-M45mutRHIM (**g**, *n* = 8 for P-RIPK3, *n* = 9 for P-MLKL) in mouse footpads with the indicated genotypes using ImageJ (Fiji) software. All data are pooled from three independent experiments. For **a**–**d**,**f**,**g**, data are presented as the mean ± s.e.m. Statistical analyses were performed using two-tailed unpaired *t*-test (**f**,**g**) or two-way ANOVA (**a**–**d**). Scale bars, 20 μm (**f**,**g**) or 50 μm (**e**). Source data

**Extended Data Fig. 6:**
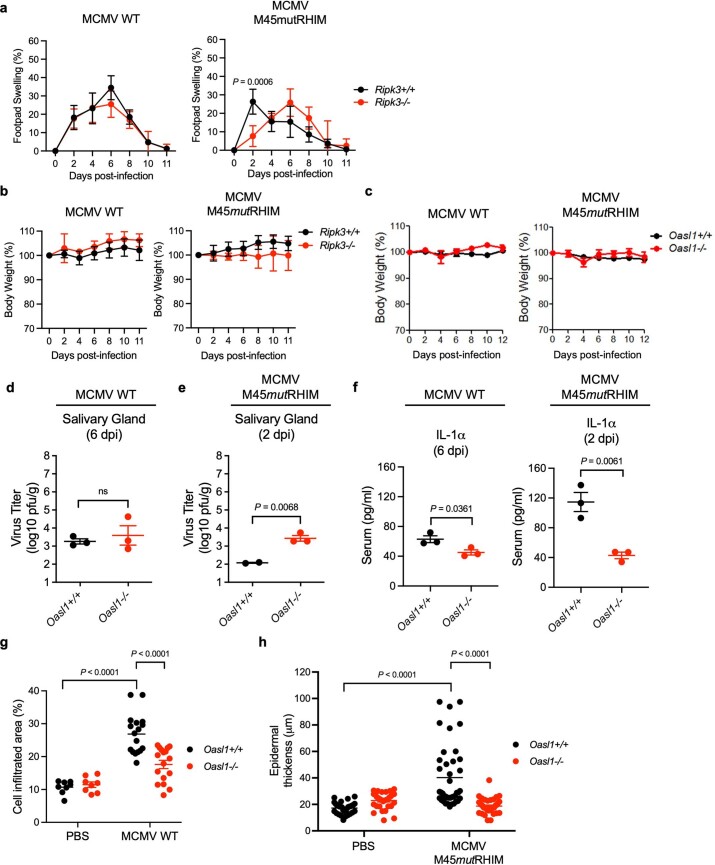
Murine OASL1 is required for MCMV infection-induced necroptosis to restrict viral dissemination *in vivo*. **a**, Measurement of footpad swelling caused by subcutaneous footpad injection of *Ripk3*^+/+^ or *Ripk3*^*-/-*^ mice with 10^6^ PFU (left) MCMV-WT (*n* = 4 per genotype) or (right) MCMV-M45*mut*RHIM (*Ripk3*^+/+^: *n* = 5, except *n* = 3 for 6 d.p.i.; *Ripk3*^*-/-*^: *n* = 5 for 0-4, 10 d.p.i., *n* = 3 for 6,8,11 d.p.i.). Data are represented as the percent increase in thickness relative to the pre-injection measurement and plotted for mean values at the indicated times. **b,c**, Body weight changes of **a** *Ripk3*^+/+^ and *Ripk3*^-/-^ mice infected with MCMV-WT (*n* = 4 per genotype) and MCMV-M45*mut*RHIM infection (*n* = 5 per genotype) and **Fig. 6a,b** *Oasl1*^+/+^ and *Oasl1*^*-/-*^ littermate mice infected with MCMV-WT (*n* = 8 for *Oasl1*^+/+^ and *n* = 6 for *Oasl1*^*-/-*^) or MCMV-M45*mut*RHIM (*n* = 8 mice per genotype). **d**,**e**, Viral titers in salivary glands (SG) from *Oasl1*^+/+^ and *Oasl1*^*-/-*^ littermate mice infected with (**d**) MCMV-WT (*n* = 3 mice per genotype) or (**e**) MCMV-M45*mut*RHIM (*n* = 2 for *Oasl1*^+/+^ and *n* = 3 for *Oasl1*^*-/-*^*)* at the indicated days post-footpad subcutaneous injection. **f**, IL-1α levels in the sera of *Oasl1*^+/+^ and *Oasl1*^*-/-*^ mice infected with MCMV-WT or MCMV-M45*mut*RHIM at the indicated days were determined by ELISA (*n* = 3 mice per genotype). **d-f**, Each symbol represents one mouse, horizontal lines represent the mean value. **g,h**, Quantification of Fig. 6e cell infiltration (*n* = 8 per genotype for PBS, *n* = 17 per genotype for MCMV-WT) and footpad epidermal thickness (*n* = 31 per genotype for PBS, *n* = 41 per genotype for MCMV-M45*mut*RHIM) in mice infected with MCMV-WT or MCMV-M45*mut*RHIM at 2 or 6 d.p.i., respectively. All data are pooled from three independent experiments. **d-h** Data are presented as mean ± SEM. Statistical analyses were performed using a two-tailed unpaired *t*-test (**d-f**) or two-way ANOVA (**a,g,h**). Source data

To further determine the role of OASL1 in virus-induced necroptotic cell death in vivo, we infected age-matched and sex-matched *Oasl1*^+/+^ and *Oasl1*^*–/–*^ littermate mice with either MCMV-WT or MCMV-M45mutRHIM via footpad injection and monitored footpad swelling for up to 12 days post infection (d.p.i.) (Fig. 6a,b). As previously described^5,6^, footpad swelling in MCMV-WT-infected *Oasl1*^+/+^ mice peaked at 6 d.p.i. (Fig. 6a), whereas it was apparently reduced in MCMV-M45mutRHIM-infected *Oasl1*^+/+^ mice (Fig. 6b). By contrast, *Oasl1*^*–/–*^ mice exhibited reduced footpad swelling upon either MCMV-WT or MCMV-M45mutRHIM infection compared with *Oasl1*^+/+^ littermates (Fig. 6a,b). Although *Ripk3*^*–/–*^ mice exhibited slightly reduced footpad swelling than WT mice upon MCMV-WT infection, they showed peak footpad swelling at 6 d.p.i. upon either MCMV-WT or MCMV-M45mutRHIM infection (Extended Data Fig. 6a,b). None of the infected mice exhibited lethality or significant body weight changes following either MCMV-WT or MCMV-M45mutRHIM infection (Extended Data Fig. 6b,c).

Previously, it has been reported that MCMV can be disseminated from the initial footpad inoculation site to the distal organ salivary glands (SGs) to establish persistent infection^58^. MCMV-WT viral titres in SGs were comparable between *Oasl1*^+/+^ and *Oasl1*^*–/–*^ mice at 6 d.p.i. (Extended Data Fig. 6d), but were significantly higher in *Oasl1*^*–/–*^ mice than in *Oasl1*^+/+^ mice at 12 d.p.i. (Fig. 6c). Notably, the dissemination of MCMV-M45mutRHIM into SGs was detected in *Oasl1*^*–/–*^ mice as early as 2 d.p.i. (Extended Data Fig. 6e), and their titres were considerably higher in *Oasl1*^*–/–*^ mice than in *Oasl1*^+/+^ mice at 12 d.p.i. (Fig. 6c). These results demonstrate that OASL1-mediated necroptosis restricts MCMV replication and dissemination.

As a consequence of necroptosis, levels of released interleukin-1α (IL-1α) in the serum were considerably higher in *Oasl1*^+/+^ mice infected with either MCMV-WT or MCMV-M45mutRHIM compared with those in *Oasl1*^*–/–*^ mice (Fig. 6d and Extended Data Fig. 6f). Consistent with the reduced footpad swelling and serum IL-1α levels, MCMV-WT-infected or M45mutRHIM-infected *Oasl1*^*–/–*^ mice showed a marked reduction in immune cell infiltration and epidermal hyperplasia (Fig. 6e and Extended Data Fig. 6g,h). Indeed, immunohistochemistry of virus-infected mouse footpads showed intense phospho-RIPK3 and phospho-MLKL signals in the dermis region of the footpads from MCMV-WT-infected or MCMV-M45mutRHIM-infected *Oasl1*^+/+^ mice, whereas the signals were substantially reduced in *Oasl1*^*–/–*^ mice (Fig. 6f,g), which suggests that OASL1 positively regulates RIPK3 and MLKL activation. Collectively, the markedly reduced activation of the key necroptotic kinase RIPK3 and the effector MLKL upon virus infection in *Oasl1*^*–/–*^ mice suggests that OASL1 has a pivotal role in the execution of necroptosis in vivo to restrict MCMV replication and elicit antiviral inflammatory responses.

### OASL1-driven necroptosis promotes antiviral activity during other viral infections

**Fig. 7:**
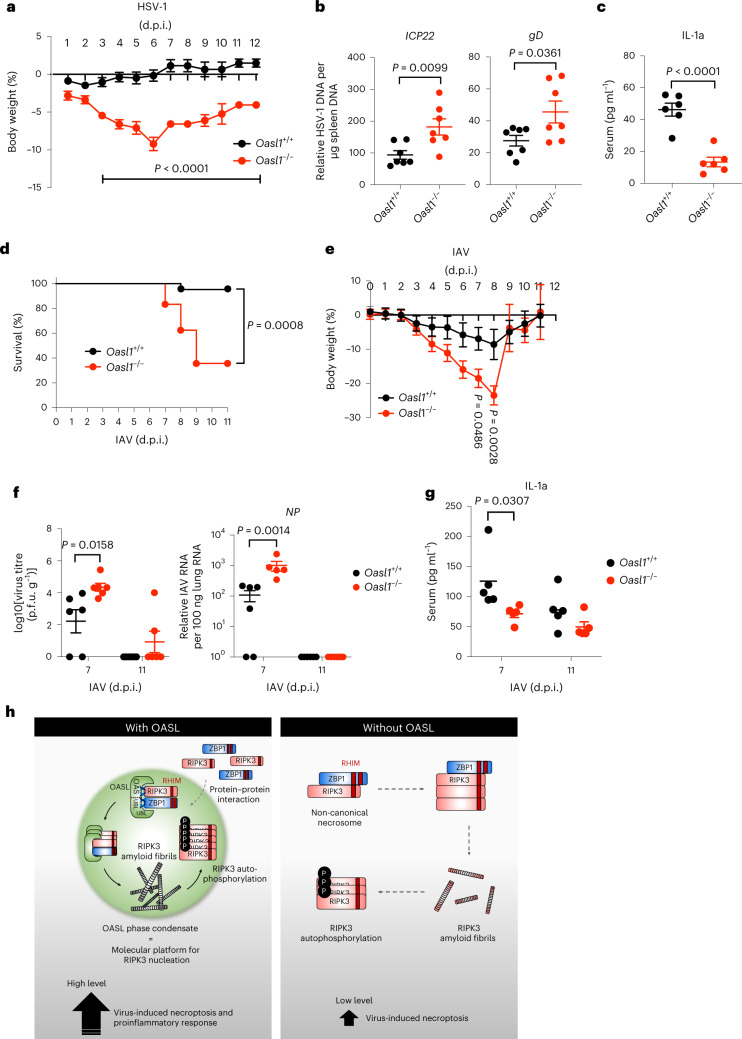
OASL1-driven necroptosis promotes antiviral activity during other viral infections. **a**, Body weight changes of age-matched and sex-matched *Oasl1*^+/+^ (*n* = 8 for 0–6 d.p.i., *n* = 4 for 7–12 d.p.i.) and *Oasl1*^*–/–*^ (*n* = 6 for 0–6 d.p.i., *n* = 2 for 7–8 d.p.i., *n* = 4 for 9–12 d.p.i.) littermate mice after intraperitoneal injection of 10^7^ p.f.u. of the HSV-1 strain KOS. **b**, Viral DNA in spleens from HSV-1-infected *Oasl1*^+/+^ and *Oasl1*^*–/–*^ littermate mice were determined by genomic DNA quantitative PCR (*n* = 7 mice per genotype). Data were normalized against a host housekeeping gene. **c**, IL-1α levels in the sera of *Oasl1*^+/+^ and *Oasl1*^*–/–*^ mice infected with HSV-1 were determined by ELISA (*n* = 5 mice per genotype). **d**, Survival analysis of age-matched and sex-matched *Oasl1*^+/+^ and *Oasl1*^*–/–*^ littermate mice intranasally infected with 100 p.f.u. of the IAV strain PR8 (*n* = 11 for *Oasl1*^+/+^, *n* = 14 for *Oasl1*^*–/–*^). **e**, Body weight changes of mice (*Oasl1*^+/+^, *n* = 10 for 0–6 d.p.i., *n* = 6 for 7–9 d.p.i., *n* = 3 for 10–11 d.p.i.; *Oasl1*^*–/–*^, *n* = 14 for 0–6 d.p.i., *n* = 11 for 7–8 d.p.i., *n* = 2 for 9–11 d.p.i.) in **d**. **f**, Virus titres and viral RNA loads in lungs from IAV-infected *Oasl1*^+/+^ and *Oasl1*^*–/–*^ littermate mice were determined by plaque assay (left) and quantitative PCR with reverse transcription (right) (*n* = 6 mice per genotype). **g**, IL-1α levels in the sera of *Oasl1*^+/+^ and *Oasl1*^*–/–*^ mice infected with IAV were determined by ELISA (*n* = 5 mice per genotype). All data are pooled from two independent experiments. For **a**–**c**,**e**–**g**, data are presented as the mean ± s.e.m. Statistical analyses were performed using two-tailed unpaired *t*-test (**b**,**c**) or two-way ANOVA (**a**,**e**–**g**). Data for **d** are presented as a Kaplan–Meier plot. For **b**,**c** and **f**,**g**, each symbol represents one mouse, and horizontal lines represent the mean value. **h**, Representative model of OASL-mediated virus-induced necroptosis. OASL undergoes LLPS and recruits RIPK3 and ZBP1 via protein–protein interactions to scaffold the assembly. OASL phase condensation induces RIPK3 nucleation and amyloid-like fibril formation, which in turn leads to RIPK3 autophosphorylation. Consequently, activated RIPK3 induces high levels of necroptosis and proinflammatory responses during virus-induced necroptosis. Source data

**Extended Data Fig. 7:**
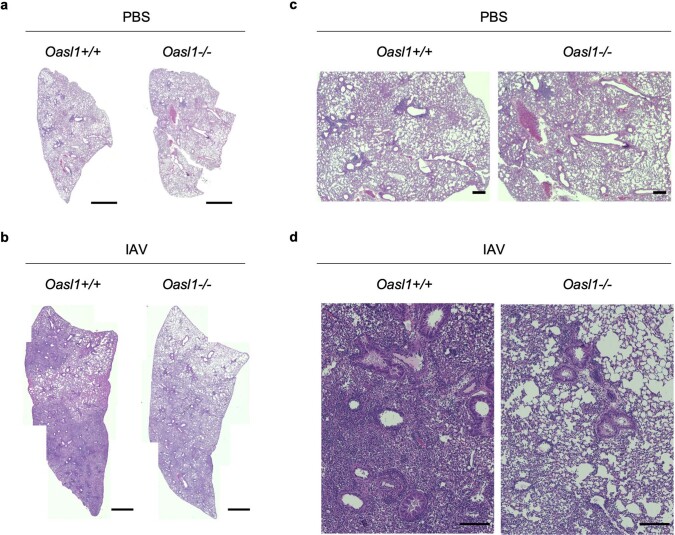
OASL1 is required for influenza virus infection-induced immune cell infiltration. **a,b**, H&E staining of whole lung sections of *Oasl1*^+/+^ and *Oasl1*^*-/-*^ littermate mice intranasally-inoculated with (**a**) PBS or (**b**) 100 PFU of IAV PR8 at 7 d.p.i. Scale bar, 1 mm. **c,d**, Representative histology of lung sections of **a,b**, respectively. Scale bar, 200 μm.

The in vivo role of OASL1 in virus-induced necroptosis was further tested for HSV-1 and IAV infection, both of which are known to induce RIPK3-mediated necroptosis^7–9,42,59^. *Oasl1*^+/+^ and *Oasl1*^*–/–*^ mice were intraperitoneally infected with 10^7^ plaque-forming units (p.f.u.) of the HSV-1 KOS strain (Fig. 7a–c) or intranasally infected with a sublethal dose of 10^2^ p.f.u. of the mouse-adapted IAV A/PR/8/H1N1 strain (Fig. 7d–g). Upon HSV-1 infection, *Oasl1*^*–/–*^ mice showed considerable weight loss compared with *Oasl1*^+/+^ mice, with the most significant reduction observed at 6 d.p.i. (Fig. 7a). Consistently, *Oasl1*^*–/–*^ mice showed significantly higher HSV-1 DNA copy number and markedly lower serum IL-1α level compared with *Oasl1*^+/+^ mice (Fig. 7b,c). Around 70% (10 out of 14) of *Oasl1*^*–/–*^ mice died from the sublethal dose of IAV infection, whereas only 9% (1 out of 11) of *Oasl1*^+/+^ littermate mice died (Fig. 7d). Similar to HSV-1 infection, more significant weight loss was observed in IAV-infected *Oasl1*^*–/–*^ mice compared with *Oasl1*^+/+^ mice, with higher peak weight loss between 6 and 8 d.p.i. (Fig. 7e). Furthermore, measuring progeny IAV production in the lungs showed 10-fold to 100-fold higher IAV titres in *Oasl1*^*–/–*^ mice compared with *Oasl1*^+/+^ mice at 7 d.p.i. (Fig. 7f). This result highlighted a correlation between mortality and greater body weight loss and higher virus burden. IAV-infected *Oasl1*^*–/–*^ mice also showed alleviated pulmonary inflammation and lower serum IL-1α levels compared with IAV-infected *Oasl1*^+/+^ littermate mice (Fig. 7g and Extended Data Fig. 7a–d). Notably, similar phenotypes have been observed when *Ripk3*^*–/–*^ mice are infected with MCMV-WT, MCMV-M45mutRHIM, HSV-1 or IAV^5,7,9,42^. Overall, these results show that OASL induces necroptosis to promote antiviral inflammation and consequently restricts viral replication and pathogenesis.

## Discussion

Robust activation of necroptosis during virus infection requires contemporaneous engagement of intact TNF and type I IFN signalling, which suggests that there is a node of pathway crosstalk between the two signalling pathways. Here we demonstrated that the IFN-inducible protein OASL is a previously undescribed component of the non-canonical necrosome complex that governs a sequential event during virus-induced necroptosis. Our study revealed that OASL undergoes dsRNA-dependent LLPS, which serves as a scaffold to facilitate the assembly of the RIPK3–ZBP1 necrosome during virus infection. These OASL liquid droplets effectively recruit RIPK3 and ZBP1, providing a spatially partitioned platform for the amyloidogenic protein RIPK3 to nucleate and induce amyloid fibril formation and prompt activation during immediate challenges (Fig. 7h). Subsequently, activated RIPK3 phosphorylates MLKL to execute high levels of virus-induced necroptosis and proinflammatory responses. A rigorous structural study is needed to further characterize the formation, as well as its functionality as molecular platforms, of the RIPK3 amyloid fibrils during virus-induced necroptosis.

Biomolecular condensates formed by LLPS through multivalent protein–nucleic acid interactions enable spatially segregated molecular platforms to fine-tune intricate biological signalling. The physical properties of droplet-like phase separation of RNA-binding proteins is not solely dependent on the negative charge of its target RNAs^60,61^. We found that OASL formed larger phase-separated condensates following treatment with poly(I:C) HMW than with poly(I:C) LMW in vitro, which implies that the length of dsRNA, and hence the multivalency, could be an important factor that determines the magnitude of OASL droplet formation^61^. Likewise, the availability of cytosolic viral dsRNA during virus infection may determine the duration and frequency of OASL LLPS. The identification of specific target dsRNA in the future can provide insights into our understanding of not only how the assembly of OASL is spatially patterned with viral dsRNA but also what secondary structure of target RNAs may trigger the necroptotic activity of OASL^60^.

Although RIPK3 is not able to undergo phase separation on its own, it has been reported that RIPK3 can heterocomplex with its RHIM-containing adaptor molecules, RIPK1 or ZBP1, to form amyloid-like structures in vitro, ultimately forming an energetically favourable conformation^18^. These RHIM–RHIM hetero-amyloids display high energetic preference over RIPK3 homo-amyloids, thereby implicating RIPK3 hetero-amyloids for a broader range of signalling activities before the formation of homo-amyloids^19^. These findings collectively suggest that an efficient assembly of higher-order signalling platforms, such as amyloid-like fibrous structures, often requires scaffolding molecules that facilitate the polymerization of subunits to become energetically favourable and consequently execute condition-specific signals. Here we showcased OASL liquid droplets that serve as a molecular platform to provide a spatial segregation region for RIPK3 to induce polymerization and amyloid-like fibril formation. Our model suggests that the OASL–RIPK3 interaction enhances the formation of the RIPK3 amyloid-like fibril signalling complex, which prolongs RIPK3 activation and drives high levels of MLKL phosphorylation, ultimately inducing robust necroptosis during virus infection. As OASL nucleates RIPK3, together with ZBP1 via RHIM-mediated interactions, within its liquid droplet, stronger intermolecular and intramolecular interactions within the droplets may allow RIPK3 to promptly achieve the minimal binding proximity and concentration for homo-oligomerization, triggering the formation of amyloid fibrils. These polymeric platforms may then exert augmented forces to trigger efficient signal transduction activity. Indeed, higher-order assemblies like amyloid fibrils have gained attention as a platform to induce signal transduction, such as apoptosis-associated spec-like protein (ASC)-dependent inflammasome assembly or FAS-associated death domain protein (FADD) and caspase-8 in apoptosis^62,63^. In-depth study of the structural perspectives of OASL liquid phase condensates is needed to elucidate how this RIPK3–ZBP1–OASL necrosome regulates condition-specific signal transduction activity.

In summary, this study elucidated a previously uncharacterized mechanism of RIPK3 activation during virus-induced necroptosis and identified a vital role of the IFN-inducible protein OASL in exploiting phase separation to activate executioner signals by scaffolding the assembly of RIPK3 and ZBP1. OASL-mediated necroptosis is an effector pathway of the IFN-mediated antiviral response that reduces virus burden by initiating proinflammatory cell death: necroptosis. This pathway is functionally distinct from the previously described role of the OAS protein family in the 2-5A synthetase-directed RNase L pathway as well as the role of OASL in IFN production. Although OASL has been implicated in differential aspects of antiviral immunity, herein we described an antiviral mechanism of OASL by defining the biochemical characteristics of OASL. This crucial role of OASL in the crosstalk between the OAS family proteins and necroptosis brings insights into our understanding of its function as a regulator of necroptosis-mediated inflammatory response to combat pathogenic challenges.

## Methods

All experiments performed in this study were approved by the Institutional Animal Care and Use Committee and the Institutional Biosafety Committee of the University of Southern California and the Cleveland Clinic.

### Primary tail fibroblasts

Primary tail fibroblasts were isolated from tails of 3–4-week-old mice and used for experiments within six passages. In brief, tails were minced and digested with 5 mg ml^–1^ STEMxyme 1 collagenase/neutral protease (Dispase, Worthington Biochemical) at 37 °C for 1 h. After transferring to complete DMEM medium for overnight culture, cell debris was filtered through a 70-µm strainer. Primary fibroblasts were then collected by centrifugation and cultured in DMEM supplemented with 10% FBS and 1% penicillin and streptomycin.

### Viruses and virological assays

MCMV-WT and MCMV-M45mutRHIM viruses^5^ were gifts from J. W. Upton (Auburn University). Viruses were propagated, concentrated and titrated by plaque assay in NIH3T3 cells. A tenfold serial dilution of whole SG homogenates were prepared in DMEM containing 2% FBS. Cells were incubated with 1 ml of serially diluted tissue homogenates at 37 °C for 2 h. The HSV-1 strain KOS was propagated and titrated by plaque assay in Vero cells.

### Mass spectrometry analysis of RIPK3-interacting proteins

Immunoprecipitation products were separated by SDS–PAGE and fixed overnight before silver staining. Protein bands were excised and sent to the Taplin Biological Mass Spectrometry Facility at Harvard Medical School for protein identification.

### Mice

Mice were bred and housed in specific pathogen-free facilities that exclude many pathogenic and adventitious viruses, bacteria and parasites, with controlled temperature (20–26 °C), illumination (10 h light–14 h dark) and humidity (30–70%) at the University of Southern California Animal Research Center at Keck Medical School and the Lerner Research Institute Biological Resources Unit at Cleveland Clinic. *Oasl1*^*–/–*^ mice were generated using the CRISPR–Cas9 system. For production of single guide RNA (sgRNA), a bicistronic vector expressing Cas9 and sgRNA^64^ were digested with BsmBI, and the linearized vector was gel-purified. A pair of oligonucleotides for target exon 2 of the mouse *Oasl1* gene (Supplementary Fig. 1a) was annealed, phosphorylated and ligated into the linearized vector. The T7 promoter was then added to the sgRNA template by PCR amplification. The gel-purified T7-sgRNA PCR product was used as the template for in vitro transcription using a MEGAshortscript T7 kit (Life Technologies). The product was subsequently purified using a MEGAclear kit (Life Technologies) and eluted in RNase-free water. Microinjection of the oligonucleotides was performed at the University of Southern California transgenic/knockout rodent core facility. In brief, *cas9* mRNA (TriLink) and sgRNA were mixed and injected into pronuclei-stage zygotes obtained from embryo donor C57BL/6 female mice. The injected zygotes were transferred into the uterus of pseudopregnant B6D2F1 (C57BL/6 × DBA2) foster female mice. For genotyping of pups, genomic DNA was extracted from the tail, and the sgRNA target site was PCR-amplified using the following primers: forward: 5′-CTACCATGACAATAAATCTCC-3′; reverse: 5′-TCTTTAGTAGTTCATTCTGCTC-3′. The PCR products were then subjected to T7E1 assays and Sanger sequencing. The protocols for performing mice studies were reviewed and approved by Institutional Animal Care and Use Committees at the University of Southern California Keck Medical School and the Cleveland Clinic.

### Plasmids, lentivirus propagation and transductions

Plasmids expressing 3′ V5-tagged mouse OASL1 and OASL2 were produced as previously described^32^ and provided by S. N. Sarkar (University of Pittsburgh Cancer Institute). Plasmid expressing HA-tagged human RIPK3 was purchased from Addgene (78804). HA-tagged N-terminal kinase domain (RIPK3_1–313_) or C-terminal unstructured region (RIPK3_310–486_) of mouse RIPK3 were generated by PCR using pcDNA3-HA-RIPK3 (Addgene, 78805) as the template and subcloning back to the same pcDNA3 background. Plasmids expressing HA-tagged mouse OASL1 and OASL1-RK mutant were generated by PCR using pcDNA3-OASL1-V5 as the template and subcloning to the lentiviral vector pCDH-MCS-EF1 (System Biosciences). To generate lentivirus, HEK 293T cells were seeded onto 6-well plates and transfected with 0.4 μg pReV, 0.4 μg pVSV and 0.8 μg pGag/Pol of the packaging vectors together with the constructs described above by using Lipofectamine 2000 (Invitrogen) according to the manufacturer’s manual. Three days after transfection, the supernatants containing lentiviruses were collected, filtered and stored at –80 °C. Primary fibroblasts were transduced with lentiviral supernatants in the presence of polybrene (8 μg ml^–1^) for 48 h and reseeded for further experiments.

### Immunoprecipitation and immunoblotting analysis

Cell lysates were prepared in lysis buffer containing 10 mM HEPES pH 7.4, 125 mM KCl and 0.5% NP-40 supplemented with protease inhibitor cocktail without EDTA (Roche), quantified by BCA, separated on SDS–PAGE gels and transferred to polyvinylidenedifluoride membranes (Bio-Rad) using a semi-dry transfer system (Bio-Rad). Primary antibody (anti-HA (BioLegend), anti-Flag (Sigma), anti-V5 (Thermo Fisher), anti-ZBP1 (Adipogen), anti-RIPK3 (Cell Signaling Technology), anti-phospho-RIPK3 (Cell Signaling Technology), anti-MLKL (Cell Signaling Technology), anti-phospho-MLKL (Cell Signaling Technology), anti-PKR (Santa Cruz), anti-actin (Santa Cruz) or anti-OASL1 (gifted by M. S. Lee, University of Ulsan College of Medicine, Korea); 1:1,000 dilution) was added and incubated overnight at 4 °C, followed by three washes in 1×PBS/0.1% Tween-20 (PBS-T). Secondary antibody (anti-rabbit, anti-mouse, anti-mouse light chain; 1:5,000 dilution) was added and incubated for 1–2 h at room temperature, followed by three washes in PBS-T. Blots were imaged using a Bio-Rad ChemiDoc Touch imaging system. For immunoprecipitation, HEK 293T cells were seeded in a 6-well plate one night before transfection with the indicated plasmids using polyethylenimine. After 48 h, cell lysates were prepared as described above, precleared by incubation with protein A/G agarose beads (Thermo) for 1 h at 4 °C and then incubated with anti-Flag (Sigma), anti-V5 (Sigma), anti-HA (Sigma) or anti-RIPK3 (Santa Cruz) antibody-conjugated beads overnight at 4 °C. After four to six washes with lysis buffer, beads containing immunoprecipitated protein complex were eluted with 2× Laemmli sample buffer (Sigma) and subjected to immunoblotting.

### Cell death analysis

For kinetic analysis of cell death, primary tail fibroblasts were seeded in 96-well clear-bottomed plates at a density to reach full confluency the next day. After overnight culture at 37 °C, cells were infected with virus at a m.o.i. of 5 for 2 h, washed out and replaced with fresh complete medium supplemented with a cell-membrane-impermeable dye, Sytox Green (0.5 μM; Thermo Fisher). Fluorescence signals of the cells were read every hour at 485 nm excitation and 535 nm emission wavelengths. Data were averaged from primary fibroblasts that were individually isolated from three to six mice with the same genotype. LDH release was determined using a CytoTox 96 Non-Radioactive Cytotoxicity Assay kit (Promega) according to the manufacturer’s manual. For ATP level analysis, CellTiter-Glo assays (Promega) were performed according to the manufacturer’s manual. Fluorescence or luminescence was recorded and analysed using a FilterMax F5 multimode microplate reader (Molecular Devices).

### Proximity Ligation Assay

PLA was performed according to the manufacturer’s instructions (Sigma). Primary tail fibroblasts were seeded at 10,000 cells per well in an 8-well chamber coverslip. After overnight incubation at 37 °C, cells were infected with MCMV-M45mutRHIM for the indicated times, washed with PBS, fixed with 4% paraformaldehyde for 15 min and permeabilized for 10 min at room temperature. The PLA pair of monoclonal rabbit RIPK3 (Cell Signaling Technology) and monoclonal mouse ZBP1 (Adipogen) antibodies were incubated overnight at 4 °C. Images were captured using a fluorescence microscope (Keyence, BZ-X710 series) and quantified using ImageJ (Fiji) software.

### Protein expression and purification

Expression constructs were generated in a pGEX-6p-1 vector to contain a PreScission-cleavable site. eGFP C-terminal fused full-length human OASL, N-OAS and C-UBL; mCherry C-terminal fused full-length human RIPK3 and C-terminal unstructured region (RIPK3_295–518_); and BFP C-terminal fused full-length human ZBP1 were expressed and purified from *Escherichia coli* strain BL21 DE3 (pLys). *E.* *coli* harbouring a glutathione *S*-transferase (GST)-tagged plasmid encoding fluorescence-fused human OASL or RIPK3 was induced by adding 0.4 mM IPTG at 16 °C for less than 16 h. Bacteria pellets were collected and lysed in lysis buffer containing 10 mM HEPES (pH 7.4), 450 mM KCl, 1 mg ml^–1^ zymolyase, 1 mM DTT, 0.1% Triton X-100 and protease inhibitor cocktail without EDTA (Roche) at room temperature for 1 h and at 4 °C for 4 h, followed by centrifugation at 16,000*g*, 4 °C for 30 min. RNase A (1 mg ml^–1^) was supplemented in the lysis buffer for human ZBP1–BFP protein purification. Supernatant was collected for overnight purification using glutathione sepharose 4B beads (Cytiva). The GST tag was removed using PreScission protease (GE Healthcare), incubating at 4 °C for 18 h. The protein concentration of the fractions containing fluorescence-tagged proteins were measured by SDS–PAGE with BSA standards.

### In vitro phase-separation assay

Purified fluorescence-tagged protein in 450 mM salt buffer was diluted to 125 mM KCl in 10 mM HEPES, pH 7.4, in 96-well plates. The plates were incubated at 37 °C and images were captured at indicated time points after incubation. All phase separation of purified protein was performed in phase-separation buffer (10 mM HEPES, pH 7.4, 125 mM KCl) without adding any crowding reagents. For RIPK3_295–518_–mCherry and ZBP1–BFP recruitment to OASL–GFP droplets, variant GFP-tagged OASL proteins, mCherry-tagged RIPK3_295–518_ and BFP-tagged ZBP1 were mixed and incubated in 96-well plates at 37 °C for 1 h at the indicated concentrations in the phase-separation buffer. Image acquisition of phase separation was performed using a fluorescence microscope (Keyence, BZ-X710 series) under ×20, ×40 and ×60 1.49-NA oil-immersion objectives. At least four independent imaging areas were analysed for each condition of each replicate. Data shown are representative of at least three independent experiments across five protein preparations.

### In vitro fibril formation assay with ThT measurement

In vitro fibril formation assays were performed as previously described^65^. In brief, 30 µM ThT (Abcam) was added to the mixtures of 0.5 μM RIPK3_295–318_–mCherry with different amounts of purified OASL and incubated at 37 °C for 16 h. ThT binding was excited using a wavelength of 430 nm and evaluated by monitoring the emission fluorescence spectrum at wavelengths from 480 nm to 550 nm.

### Electron microscopy and immunogold labelling

A total of 5 μl of individual or mixtures of OASL–GFP, RIPK3_295–518_–mCherry and ZBP1–BFP proteins were spotted onto Parafilm, and a carbon–formvar-coated copper grid (200 mesh, Ted Pella) was placed on the surface of the droplet. After 1 min of absorption at room temperature, the sample was wicked off the grid by touching the filter paper. The grids were washed with deionized water three times and stained by floating the grids on a droplet of 2% uranyl acetate. For immunogold labelling, the grids were blocked in 1% BSA in PBS for 10 min, followed by staining with rabbit monoclonal anti-RIPK3 antibody (Cell Signaling Technology) at 1:100 dilution for 2 h. Next, the grids were washed with 1% BSA in PBS for six times, followed by incubation with secondary antibodies (anti-rabbit conjugated with 6 nm and 10 nm gold particles) for 30 min. The grids were washed with PBS six times, followed by deionized water washing and stained with 2% uranyl acetate. Grids were examined and imaged using a transmission electron microscope (FEI, Tecnai G2 Spirit) at 120 kV operated by the Imaging Core of the Lerner Research Institute at the Cleveland Clinic.

### Transmission electron tomography

Carbon–formvar-coated copper grids (200 mesh, Ted Pella) were coated with 0.1% (w/v) poly-l-lysine (Sigma) for 10 min and blotted to remove residual liquid. After drying, the grids were incubated with a droplet of sample for 5 min and subsequently washed with distilled water for 5 s, followed by 2% uranyl acetate staining. Afterwards, the grids were treated with 10 nm gold particles (741957, Sigma Aldrich) in water (1:1) and blotted. Single-axis tilt electron tomography was recorded from −60° to 60° using tilt increments of 2° using a transmission electron microscope (FEI, Tecnai F20) at 200 kV equipped with a 4,096 × 4,096 pixel CMOS camera (TemCam-F416, TVIPS). The image stack was generated from tilt images and subjected to tomographic reconstructions using the weighted back-projection algorithm in IMOD (v.4.11.2). The 3D model of amyloids was manually traced from the virtual sections of the tomogram.

### In vitro kinase assay

In vitro kinase assays were performed as previously described^2,25^. Human RIPK3, kinase-dead RIPK3-K50A and phosphorylation-dead RIPK3-S227A mutant proteins were purified from *E.* *coli* using glutathione sepharose beads. After removing the GST tag, 1 ng of RIPK3 protein was mixed with different amounts of purified human OASL protein (5–50 ng) in kinase reaction buffer (20 mM HEPES, pH 7.4, 1 mM DTT, 20 mM MnCl_2_, 20 mM MgCl_2_, 1 mM EDTA and 100 μM ATP) supplemented with phosphatase inhibitor cocktail (Sigma). For RIPK3 and MLKL in vitro kinase assays, OASL–GFP and ZBP1–BFP were purified from *E.* *coli*, and GST–RIPK3 (Sigma) and MLKL (Cusabio) were commercially purchased. GST–RIPK3 (10 ng), ZBP1 (50 ng) and OASL (50 ng) were subjected to phase separation at 37 °C before kinase reaction with MLKL and the kinase reaction buffer (50 ng). The kinase reaction was performed at 30 °C for 45 min in a total volume of 20 μl, stopped with 20 μl of 2× Laemmli sample buffer, boiled for 5 min and subjected to immunoblotting analysis with the indicated anti-phospho antibodies.

### Immunostaining and confocal microscopy

Primary tail fibroblasts were seeded onto an 8-well chamber slide (Nunc Lab-Tek II Chamber Slide system) for overnight culture at 37 °C and infected with a m.o.i. = 5 of MCMV-M45mutRHIM mutant virus for the indicated times. ThT (25 μM) was added to cells 1 h before fixation^18^. Cells were fixed with 4% paraformaldehyde for 15 min, permeabilized in 0.1% Triton X-100 in PBS for 10 min and blocked with 0.5% BSA in PBS containing 0.1% saponin at room temperature. Cells were then incubated with the indicated antibodies overnight at 4 °C, followed by washing with PBS-T three times at room temperature and incubation with anti-mouse IgG Cascade Blue, anti-mouse IgG Alexa Fluor 568, anti-rabbit IgG Alexa Fluor 488 or anti-rabbit IgG Alexa Fluor 568 (Thermo Fisher) at 1:1,000 dilution. The coverslips were mounted and counterstained using ProLong Gold Antifade mountant with 4,6-diamidino-2-phenylindole (DAPI, Thermo Fisher). All images were captured using identical settings on a Nikon laser scanning confocal microscope or a Leica SP8 confocal microscope with a ×60 oil-objective, and processed using Nikon’s NIS elements, Leica’s LAS X and ImageJ software.

### Fluorescent recovery after photobleaching assay

Cellular FRAP experiments were performed using a LSM880 Airyscan microscope at 37 °C in a live-cell imaging chamber. *Oasl1*^*–/–*^ primary fibroblasts expressing OASL1–mCherry were grown on glass-bottom dishes (MatTek) overnight to reach the desired density. Cells were then inoculated with MCMV-M45mutRHIM for 2 h. OASL1–mCherry condensates were identified by live-staining with SYTO 45 (Thermo Fisher) 30 min before bleaching and partially photobleaching with 50% laser power using a 560 nm laser. Time-lapse images were acquired with 2-s intervals over 2 min after photobleaching. Images were processed using ImageJ, and FRAP data were fit to a single exponential model using GraphPad Prism. The background intensity was subtracted, and values are reported relative to pre-bleaching time points.

### Tomography and analysis of parameters

A commercial optical diffraction tomographic system with modifications for 3D fluorescence imaging (HT-2, Tomocube) was utilized to measure the RI tomograms of individual liquid-like droplets. All morphological and biochemical parameters were quantitatively obtained from RI tomograms, as previously described^66^, using the TomoStudio software.

### Infection of mice and organ collection

O*asl1*^+/+^ and *Oasl1*^*–/–*^ mice were anaesthetized with isoflurane, followed by subcutaneous injection of 10^6^ p.f.u. MCMV-WT or MCMV-M45mutRHIM virus into the ventral side of the footpads^5^. Infected mice were observed over a period of 14 days for body weight loss and survival. All experiments were performed with sex-matched mice at 6–8 weeks of age. After euthanasia, organs were collected in PBS and stored at –80 °C until thawed for plaque assays. Experiments involving mice were performed under an approved protocol from the Institutional Animal Care and Use committees at the University of Southern California Keck School of Medicine and Cleveland Clinic.

### Histological analysis of mouse tissue

Mouse footpads were collected, fixed in 4% paraformaldehyde for 24 h and transferred to 70% ethanol for storage. Tissue sectioning and immunohistochemistry were performed by the University of Southern California Immunohistochemistry Core facility. In brief, footpads were decalcified in formic acid (Immunocal, StatLab), embedded in paraffin and sectioned at 5 μm thickness. Slides were deparaffinized and either stained with haematoxylin and eosin or antigen-retrieved using retrieval buffer for immunohistochemistry staining. Anti-phospho RIPK3 (Cell Signaling Technology) and anti-phospho MLKL (Cell Signaling Technology) were used for staining. Staining was visualized using streptavidin–HRP (Millipore) and DAB substrate (DAKO and Vector Lab). All immunohistochemistry sections were counterstained with haematoxylin. Images were captured using a bright-field microscope (Keyence, BZ-X710 series). Quantification of phosphorylation signals, epidermatitis or inflamed area were calculated into percentage values (positive signal area versus total area of field of view) on individual footpad cross-sections.

### ELISA

Cytokines in serum from animal experiments were quantified using ELISA kits for mouse IL-1α (Invitrogen) according to the manufacturer’s protocol.

### Statistics and reproducibility

Statistical significance was determined using two-tailed unpaired Student’s *t*-test for two component comparisons and one-way analysis of variance (ANOVA) with Tukey’s comparison or two-way ANOVA with Bonferroni’s comparison for multi-component comparisons in GraphPad Prism v.9.1. Survival curves were generated using the Kaplan–Meier method. Quantification of area was performed using ImageJ (Fiji). All experiments were repeated as indicated in the figure legends with a minimum of two independent replications. The exact value of *n*, representing the number of mice or samples in the experiments, is indicated in each figure legend. No statistical method was used to predetermine sample sizes. Sample sizes were chosen on the basis of standard practice in the field. Data distribution was assumed to be normal, but was not formally tested. No data were excluded from the analyses. No randomization or blinding was used.

### Reporting summary

Further information on research design is available in the Nature Portfolio Reporting Summary linked to this article.

## Online content

Any methods, additional references, Nature Portfolio reporting summaries, source data, extended data, supplementary information, acknowledgements, peer review information; details of author contributions and competing interests; and statements of data and code availability are available at 10.1038/s41556-022-01039-y.

### Supplementary information

Supplementary Information Supplementary Table 1

Reporting Summary

Supplementary Video 1 **FRAP analysis of SYTO-45-stained OASL1–mCherry focus**. Representative movie of Fig. 3k. OASL1–dsRNA foci before and after photobleaching. The rectangle frame represents the bleached and recovered area within the foci. Scale bar, 10 μm

Supplementary Video 2 **FRAP analysis of SYTO-45-stained OASL1–mCherry focus**. Representative movie of Extended Data Fig. 3j. OASL1–dsRNA foci before and after photobleaching. The rectangle frame represents the bleached and recovered area within the foci. Scale bar, 10 μm.

Supplementary Video 3 **FRAP analysis of SYTO-45-stained OASL1–mCherry focus**. Representative movie of Extended Data Fig. 3k. OASL1–dsRNA foci before and after photobleaching. The rectangle frame represents the bleached and recovered area within the foci. Scale bar, 10 μm.

Supplementary Video 4 **Electron tomogram of RIPK3**_**295–518**_ **amyloid fibrils**. Representative movie of RIPK3_295–518_ of Fig. 5d. Virtual sections through a tomogram of RIPK3_295–518_–mCherry amyloid fibrils and overlay of the 3D model section. Scale bar, 50 nm.

Supplementary Video 5 **Electron tomogram of RIPK3**_**295–518**_ **amyloid fibrils**. Representative movie of RIPK3_295–518_ + ZBP1 of Fig. 5d. Virtual sections through a tomogram of RIPK3_295–518_–mCherry amyloid fibrils and overlay of the 3D model section after incubation with ZBP1–BFP. Scale bar, 50 nm.

Supplementary Video 6 **Electron tomogram of RIPK3**_**295–518**_ **amyloid fibrils**. Representative movie of RIPK3_295–518_ + OASL of Fig. 5d. Virtual sections through a tomogram of RIPK3_295–518_–mCherry amyloid fibrils and overlay of the 3D model section after incubation with OASL–GFP. Scale bar, 50 nm.

Supplementary Video 7 **Electron tomogram of RIPK3**_**295–518**_ **amyloid fibrils**. Representative movie of RIPK3_295–518_ + ZBP1 + OASL of Fig. 5d. Virtual sections through a tomogram of RIPK3_295–518_–mCherry amyloid fibrils and overlay of the 3D model section after incubation with both ZBP1–BFP and OASL–GFP. Scale bar, 50 nm.

### Source data

Source Data Fig. 1 Statistical source data.

Source Data Fig. 1 Unprocessed western blot.

Source Data Fig. 2 Statistical source data.

Source Data Fig. 2 Unprocessed western blot.

Source Data Fig. 3 Statistical source data.

Source Data Fig. 4 Unprocessed western blot.

Source Data Fig. 5 Statistical source data.

Source Data Fig. 5 Unprocessed western blot.

Source Data Fig. 6 Statistical source data.

Source Data Fig. 7 Statistical source data.

Source Data Extended Data Fig. 1 Statistical source data.

Source Data Extended Data Fig. 1 Unprocessed western blot.

Source Data Extended Data Fig. 2 Statistical source data.

Source Data Extended Data Fig. 2 Unprocessed western blot.

Source Data Extended Data Fig. 3 Statistical source data.

Source Data Extended Data Fig. 4 Statistical source data.

Source Data Extended Data Fig. 5 Statistical source data.

Source Data Extended Data Fig. 6 Statistical source data.

## Data Availability

The data that support the findings of this study are available from the corresponding author upon reasonable request. Source data are provided with this paper.
