# Supplementary material for: OASL phase condensation induces amyloid-like fibrillation of RIPK3 to promote virus-induced necroptosis
Source: Nat Cell Biol. 2023 Jan 5;25(1):92–107. doi: 10.1038/s41556-022-01039-y (PMC9859756; doi:10.1038/s41556-022-01039-y)
Supplement: Supplementary file 2 — Reporting Summary [file 41556_2022_1039_MOESM2_ESM.pdf]

nature portfolio

Corresponding author(s): Jae U. Jung

Last updated by author(s): Oct 4, 2022

Reporting Summary

Nature Portfolio wishes to improve the reproducibility of the work that we publish. This form provides structure for consistency and transparency in reporting. For further information on Nature Portfolio policies, see our Editorial Policies and the Editorial Policy Checklist.

Statistics

For all statistical analyses, confirm that the following items are present in the figure legend, table legend, main text, or Methods section.

n/a	Confirmed	
☐	☒	The exact sample size (*n*) for each experimental group/condition, given as a discrete number and unit of measurement
☐	☒	A statement on whether measurements were taken from distinct samples or whether the same sample was measured repeatedly
☐	☒	The statistical test(s) used AND whether they are one- or two-sided<br>*Only common tests should be described solely by name; describe more complex techniques in the Methods section.*
☐	☒	A description of all covariates tested
☐	☒	A description of any assumptions or corrections, such as tests of normality and adjustment for multiple comparisons
☐	☒	A full description of the statistical parameters including central tendency (e.g. means) or other basic estimates (e.g. regression coefficient) AND variation (e.g. standard deviation) or associated estimates of uncertainty (e.g. confidence intervals)
☐	☒	For null hypothesis testing, the test statistic (e.g. *F*, *t*, *r*) with confidence intervals, effect sizes, degrees of freedom and *P* value noted<br>*Give P values as exact values whenever suitable.*
☒	☐	For Bayesian analysis, information on the choice of priors and Markov chain Monte Carlo settings
☒	☐	For hierarchical and complex designs, identification of the appropriate level for tests and full reporting of outcomes
☒	☐	Estimates of effect sizes (e.g. Cohen's *d*, Pearson's *r*), indicating how they were calculated

Our web collection on statistics for biologists contains articles on many of the points above.

Software and code

Policy information about availability of computer code

Data collection	1. Immunofluorescence images were acquired using NIS-Elements Confocal (Nikon), BZ-X710 microscope software (Keyence), and LAS X (Leica).<br>2. FRAP assay was performed using Zen (Zeiss).<br>3. 3D holotomography imaging was performed using TomoStudio (Tomocube).<br>4. TEM imaging was performed using EM-MENU software (TVIPS) or Tecnai Spirit G2, FEI software.<br>5. Immunoblotting data were imaged using Chemidoc Chemiluminescence system (Bio-Rad).<br>6. qRT-PCR data were acquired using CFX Manager 3.1 software (Bio-Rad).
Data analysis	Statistical analysis was performed using Prism v9.1 (GraphPad). FRAP analysis was performed using Zen black edition v3.0 (Zeiss). Holotomography image analysis was performed using TomoStudio v2.6.15 (Tomocube). TEM image analysis was performed using IMOD (v4.11.2). All other image analyses were performed using Image J (FIJI).

For manuscripts utilizing custom algorithms or software that are central to the research but not yet described in published literature, software must be made available to editors and reviewers. We strongly encourage code deposition in a community repository (e.g. GitHub). See the Nature Portfolio guidelines for submitting code & software for further information.

Data

Policy information about availability of data

All manuscripts must include a data availability statement. This statement should provide the following information, where applicable:

- Accession codes, unique identifiers, or web links for publicly available datasets
- A description of any restrictions on data availability
- For clinical datasets or third party data, please ensure that the statement adheres to our policy

Data that support the findings of this study are available from the corresponding author upon request.

Field-specific reporting

Please select the one below that is the best fit for your research. If you are not sure, read the appropriate sections before making your selection.

☒ Life sciences ☐ Behavioural & social sciences ☐ Ecological, evolutionary & environmental sciences

For a reference copy of the document with all sections, see nature.com/documents/nr-reporting-summary-flat.pdf

Life sciences study design

All studies must disclose on these points even when the disclosure is negative.

Sample size	No statistical methods were used to predetermine sample size. Sample size was determined based on the numbers required to generate statistical power and preliminary experiments.
Data exclusions	No data were excluded from the analyses.
Replication	All experiments were independently repeated at least twice to ensure reproducibility of findings. Details are clearly indicated in each figure legend.
Randomization	All animals were assigned to groups randomly.
Blinding	Blinding was not relevant to the study.

Reporting for specific materials, systems and methods

We require information from authors about some types of materials, experimental systems and methods used in many studies. Here, indicate whether each material, system or method listed is relevant to your study. If you are not sure if a list item applies to your research, read the appropriate section before selecting a response.

Materials & experimental systems

n/a	Involved in the study	
☐	☒	Antibodies
☐	☒	Eukaryotic cell lines
☒	☐	Palaeontology and archaeology
☐	☒	Animals and other organisms
☒	☐	Human research participants
☒	☐	Clinical data
☒	☐	Dual use research of concern

Methods

n/a	Involved in the study	
☒	☐	ChIP-seq
☒	☐	Flow cytometry
☒	☐	MRI-based neuroimaging

Antibodies

Antibodies used

Human RIPK3 (Cell Signaling Technology; Cat# 13526; E1Z1D; Lot# 3)
Human p-RIPK3 (Cell Signaling Technology; Cat# 93654; D6W2T; Lot# 2)
Mouse RIPK3 (Cell Signaling Technology; Cat# 95702; D4G2A; Lot# 3)
Mouse p-RIPK3 (Cell Signaling Technology; Cat# 57220; E7S1R; Lot# 1)
Mouse MLKL (Cell Signaling Technology; Cat# 37705; D6W1K; Lot# 1)
Mouse p-MLKL (Cell Signaling Technology; Cat# 37333; D6E3G; Lot# 2)
Human OASL (Santa Cruz; Cat# sc-130832; L-25; Lot# A2214)
Mouse OASL1 (Gifted by Dr. Myeong Sup Lee)
ZBP1 (AdipoGen; Cat# AG-20B-0010-C100; Zippy-1; Lot#A28231605)
PKR (Santa Cruz; Cat# sc-707; K-17; Lot# F1214)
Actin (Santa Cruz; Cat# sc-47778; C4; Lot# J1512)

Mouse-HA (BioLegend; Cat# 901503; 16B12; Lot# B200768)
Rabbit-HA (BioLegend; Cat# 902301; PRB-101P; Lot# E11BF00)
Mouse-FLAG (Sigma; Cat# F1804; Lot# SLBR7936V)
Rabbit-FLAG (Sigma; Cat# F7425; Lot# 086M4803V)
Mouse-V5 (Thermo Fisher; Cat# R960-25; Lot# 2184509)
Rabbit Alexa Fluor 488 (Thermo Fisher; Cat# A11008; Lot# 2018309)
Rabbit Alexa Fluor 568 (Thermo Fisher; Cat# A11011; Lot# 2192277)
Mouse Alexa Fluor 568 (Thermo Fisher; Cat# A10037; Lot# 2110843)
Mouse Cascade Blue (Thermo Fisher; Cat# C962; Lot# 1316675)

Validation	Each primary antibody was validated for the species and application according to previous publications and the manufacturers' datasheet, which are available on the manufacturer website. The antibodies were further validated internally and routinely used in the lab. Data are provided and details are reported in Methods.

Eukaryotic cell lines

Policy information about cell lines

Cell line source(s)	HEK-293T, NIH3T3, Vero, and A549 cells were obtained from ATCC.<br>Primary murine fibroblasts were obtained from C57BL/6 mice.
Authentication	Cell lines were not authenticated.
Mycoplasma contamination	All cell lines were tested negative for mycoplasma contamination.
Commonly misidentified lines<br>(See ICLAC register)	No commonly misidentified cell lines were used.

Animals and other organisms

Policy information about studies involving animals; ARRIVE guidelines recommended for reporting animal research

Laboratory animals	Wild-type, Oasl1-/-, and Ripk3-/- mice are C57BL/6 genetic background. 3-4-week-old mice of equal distribution were used for primary fibroblast isolation from the tail. 6-8-week-old age mice of equal distribution were used for in vivo experiments. Details are reported in Methods.
Wild animals	Study did not involve wild animals.
Field-collected samples	Study did not involve samples collected from the field.
Ethics oversight	All mice were used in accordance with protocols and ethical regulations approved by the University of Southern California and Cleveland Clinic Institutional Animal Care and Use Committee.

Note that full information on the approval of the study protocol must also be provided in the manuscript.
