# Supplementary figures and images for: OASL phase condensation induces amyloid-like fibrillation of RIPK3 to promote virus-induced necroptosis
Source: Nat Cell Biol. 2023 Jan 5;25(1):92–107. doi: 10.1038/s41556-022-01039-y (PMC9859756; doi:10.1038/s41556-022-01039-y)

**Fig. 1b**

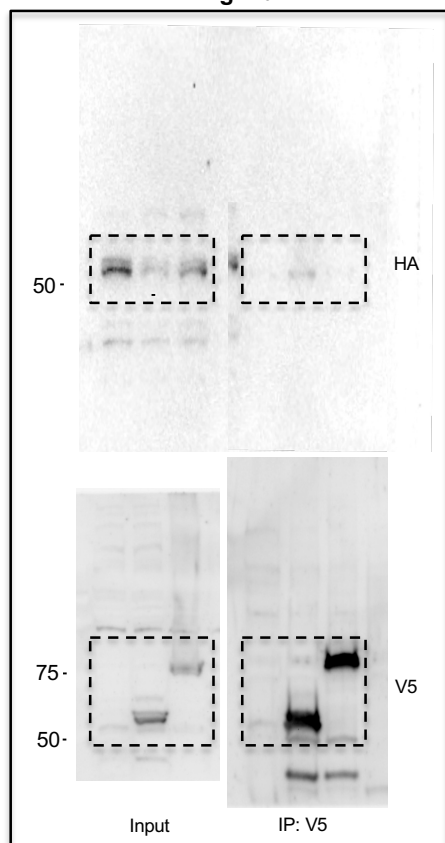

**Fig. 1f**

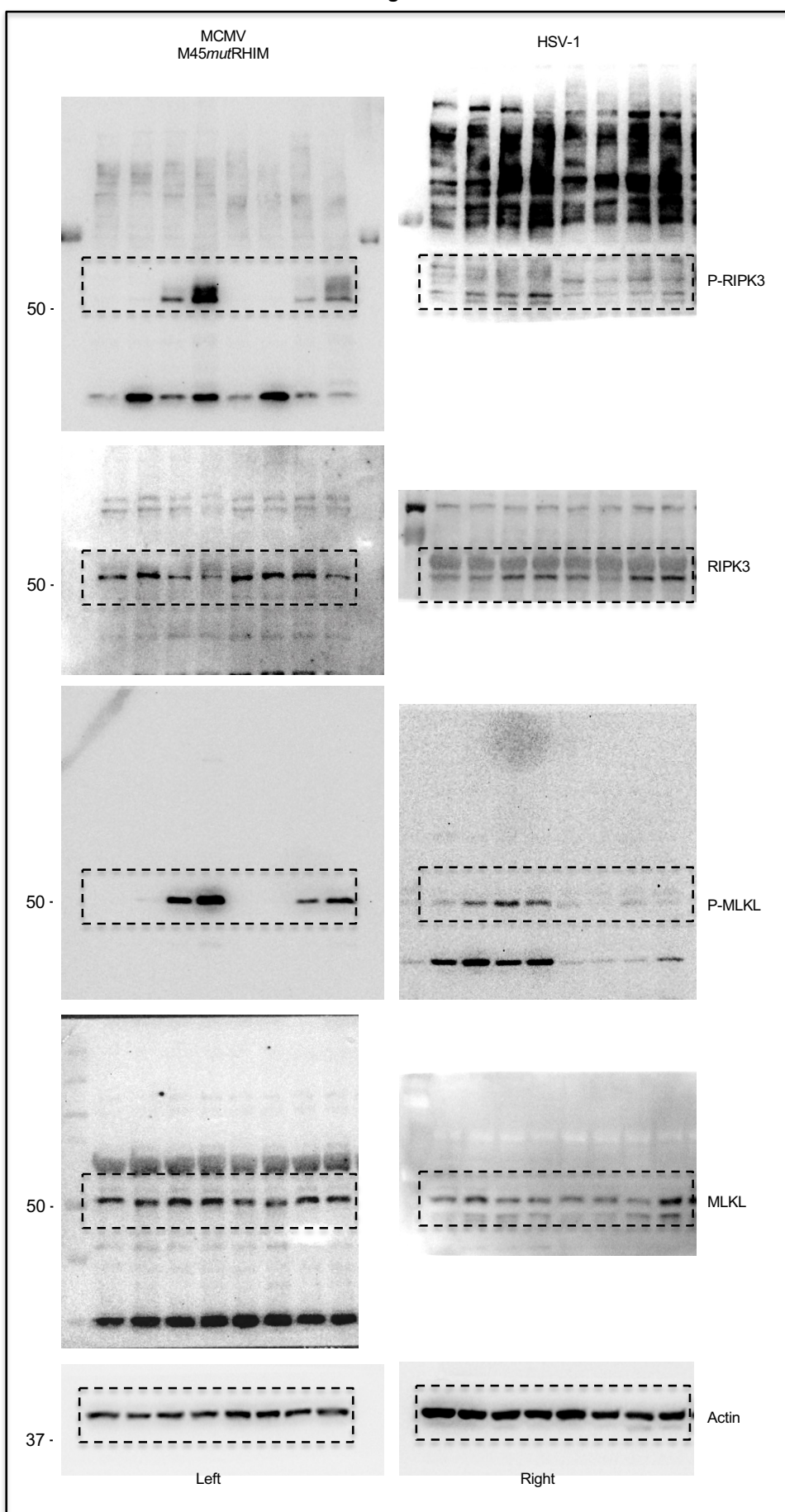

Supplement: Supplementary file 11 — Unprocessed western blot. [file 41556_2022_1039_MOESM11_ESM.pdf]

**Fig. 2a**

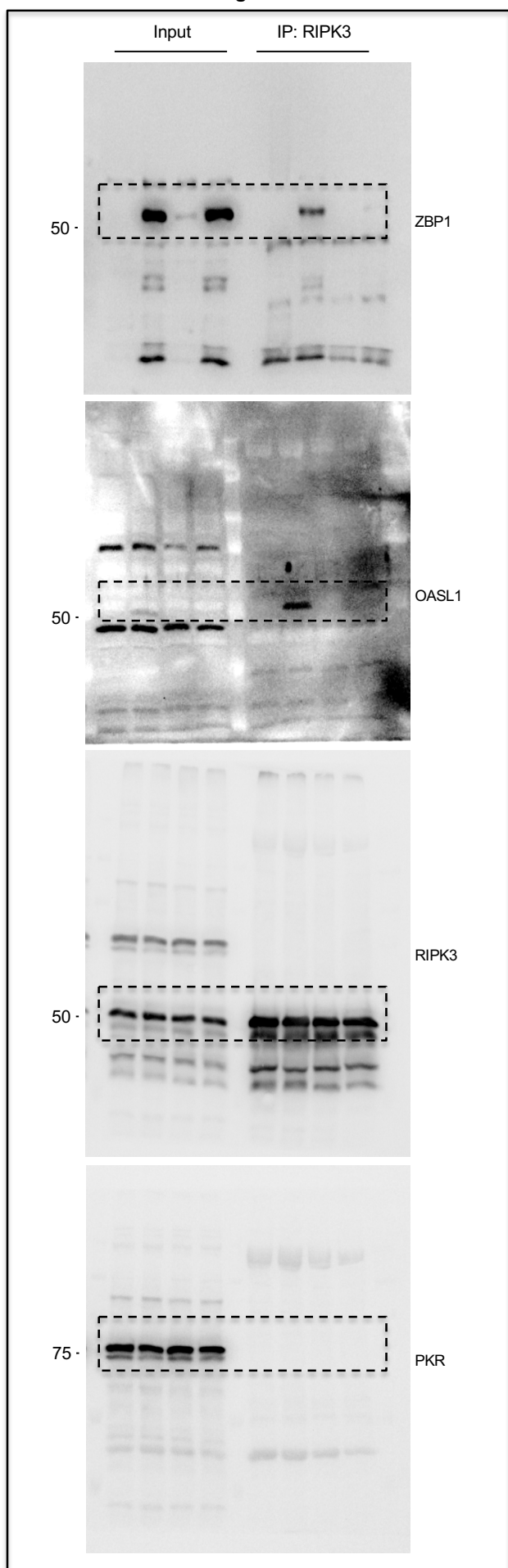

**Fig. 2d**

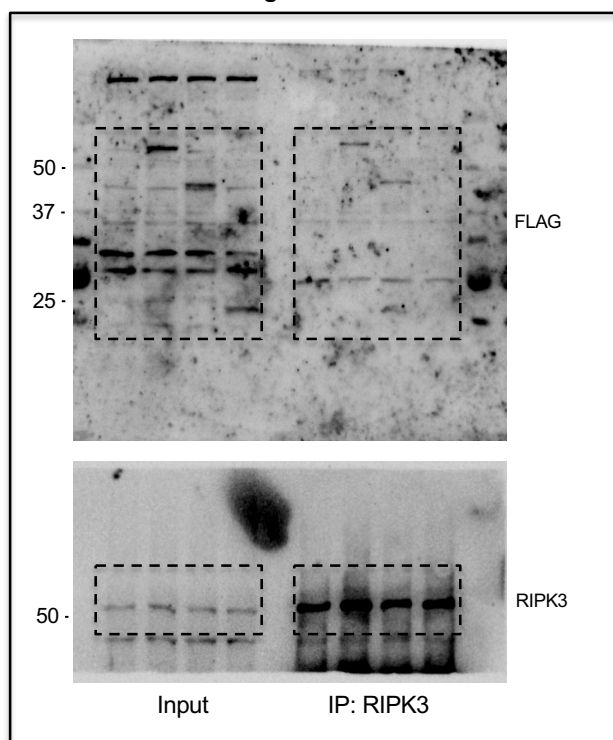

**Fig. 2e**

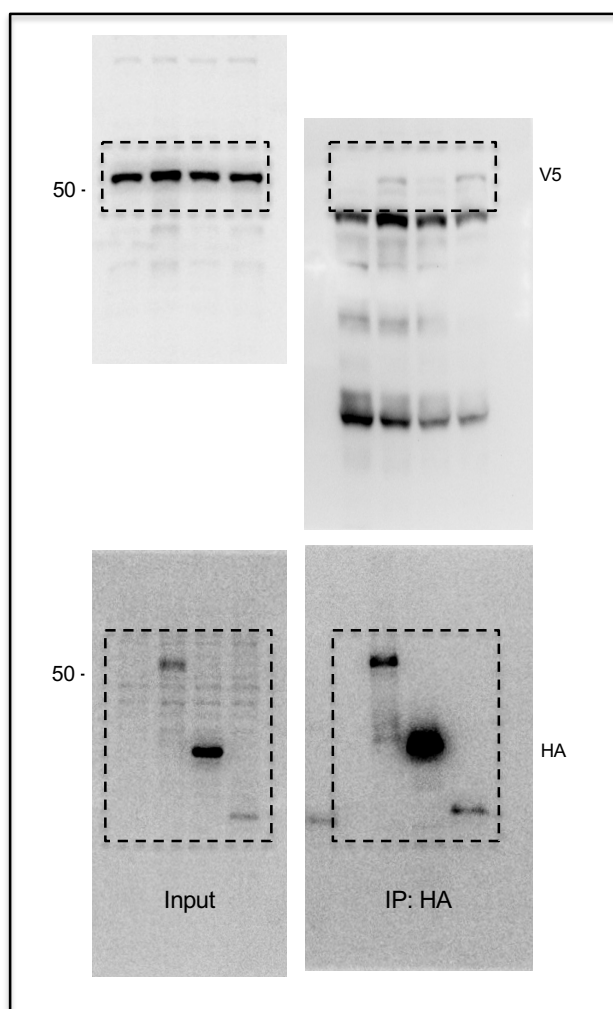

Fig. 2g

MCV  
M45mutRHIM

50

50

P-MLKL

MLKL

HSV-1

50

50

P-MLKL

MLKL

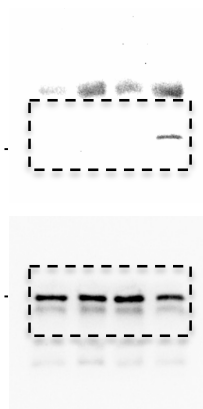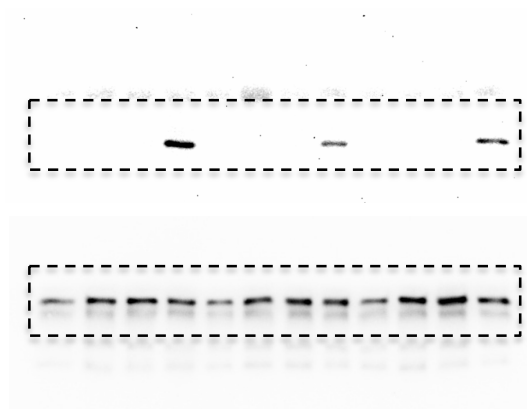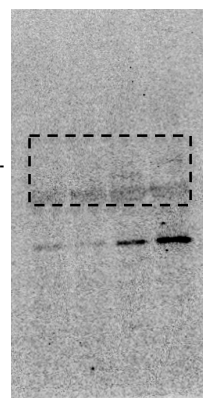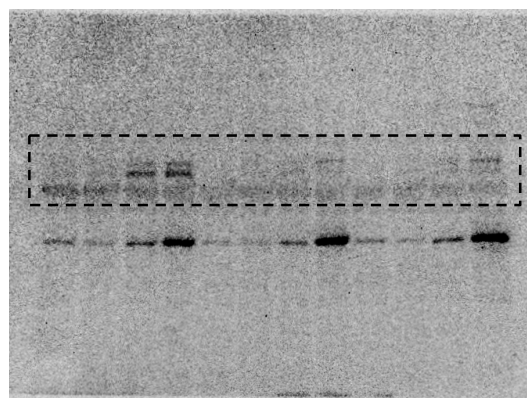

Supplement: Supplementary file 13 — Unprocessed western blot. [file 41556_2022_1039_MOESM13_ESM.pdf]

Fig. 4f

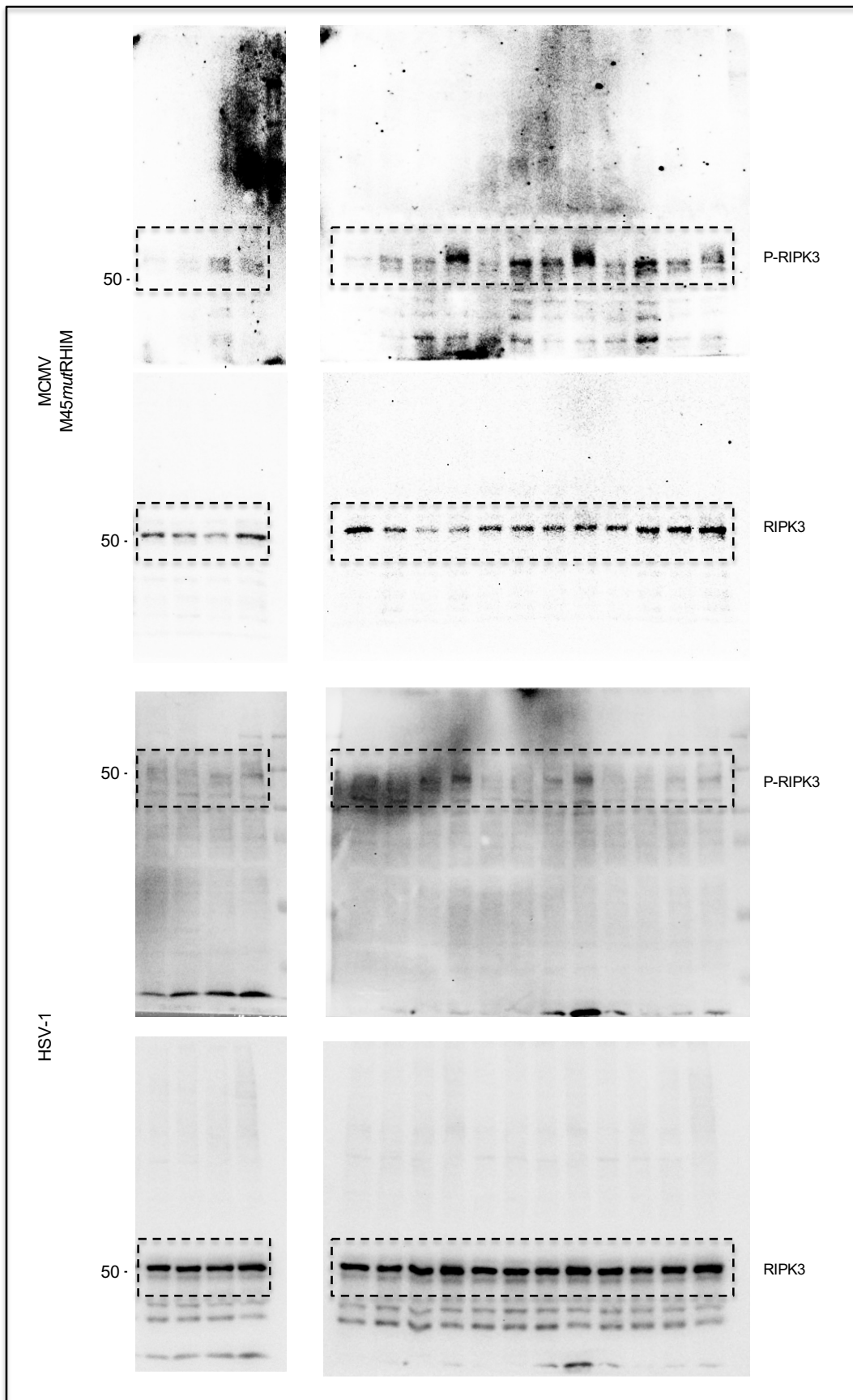

Fig. 4g

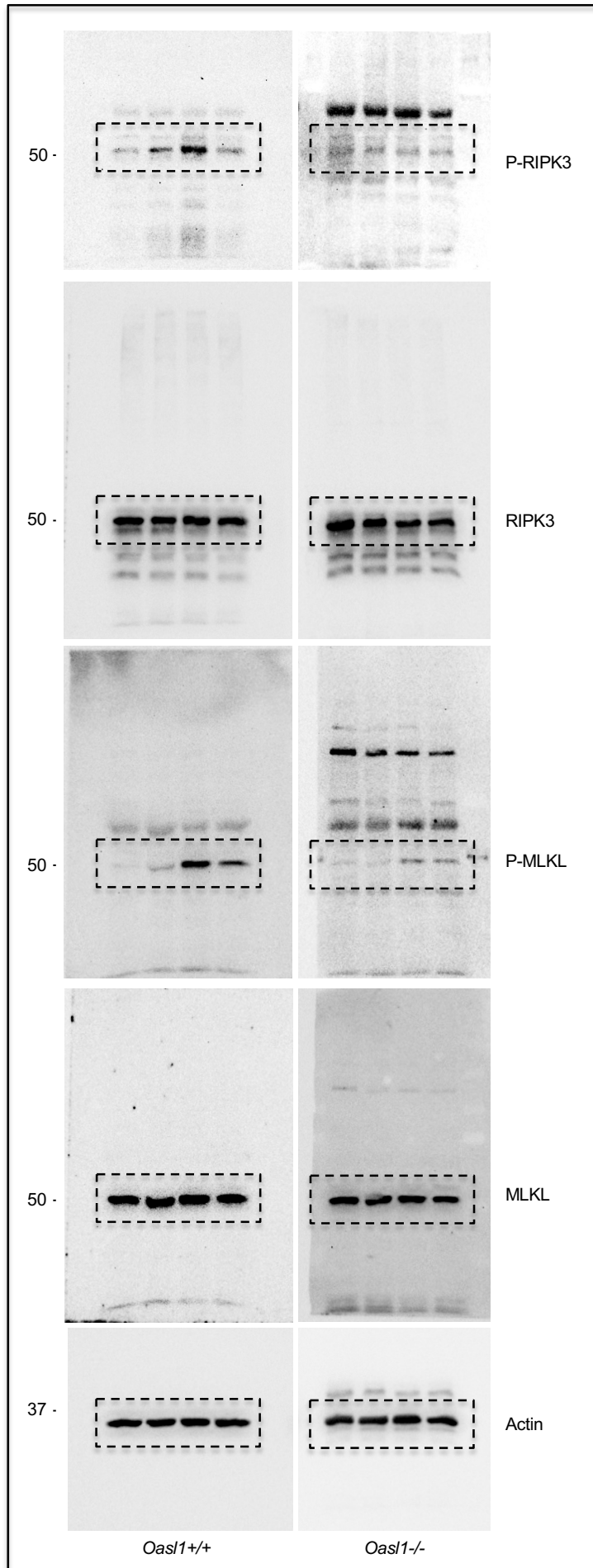

Fig. 4h

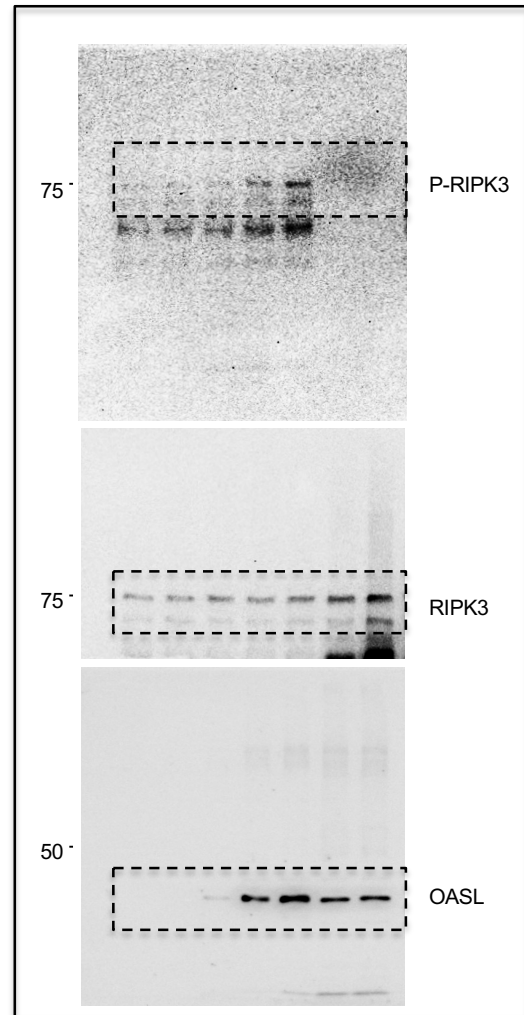

Supplement: Supplementary file 15 — Unprocessed western blot. [file 41556_2022_1039_MOESM15_ESM.pdf]

Fig. 5g

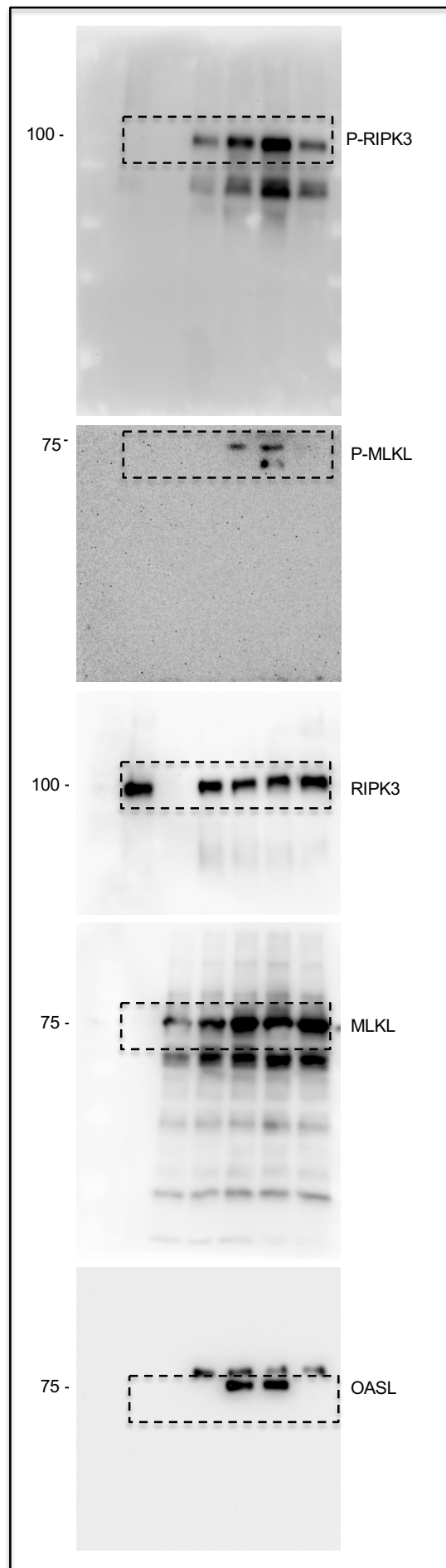

Supplement: Supplementary file 17 — Unprocessed western blot. [file 41556_2022_1039_MOESM17_ESM.pdf]

Supplementary Fig. 1c

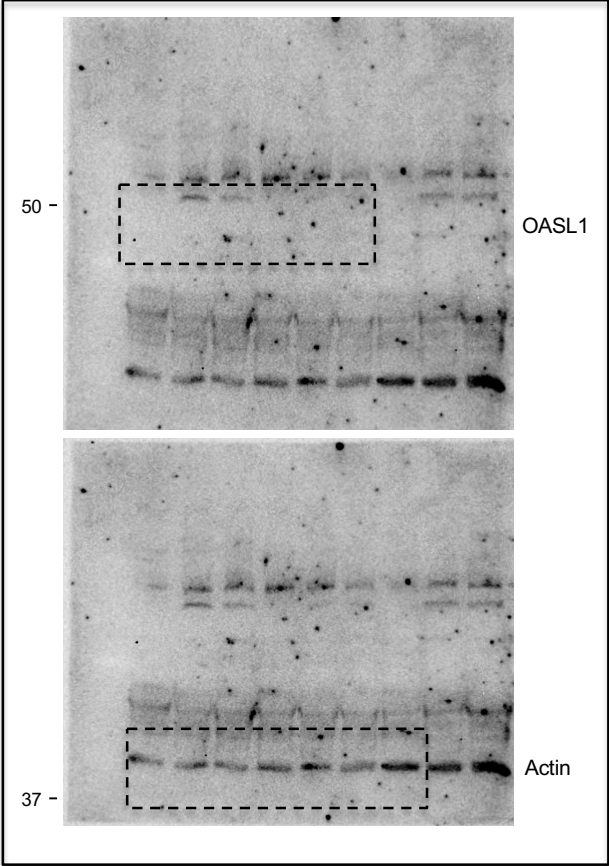

Supplementary Fig. 1g

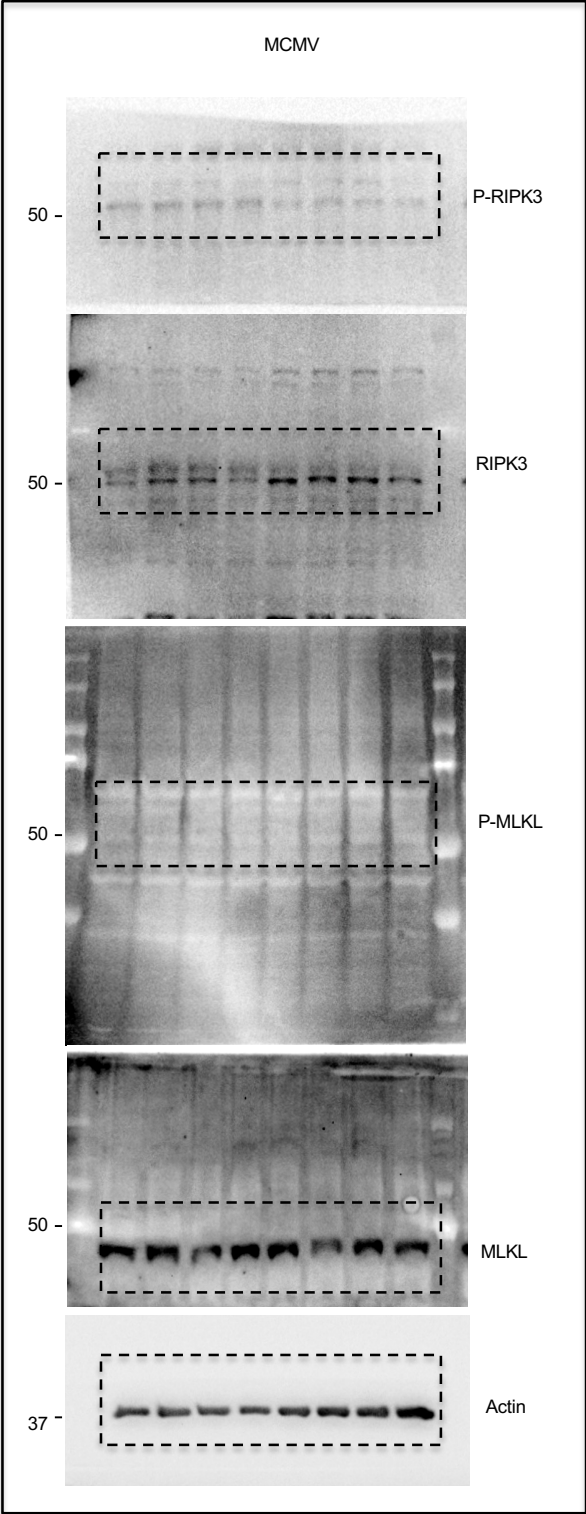

Supplementary Fig. 1d

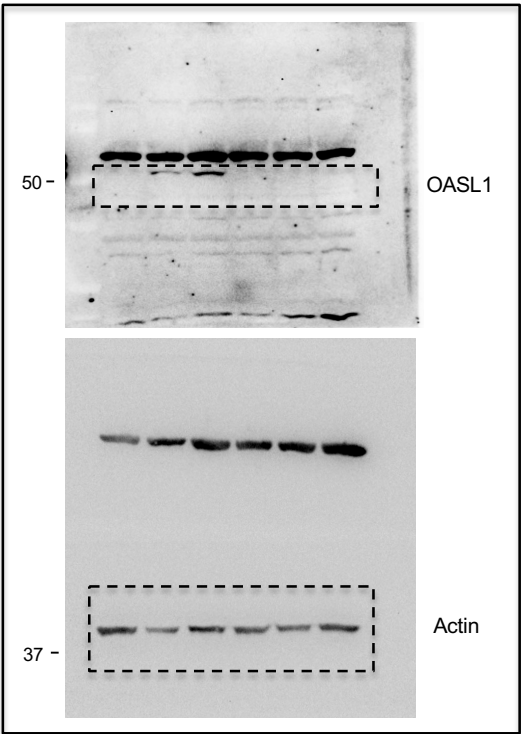

Supplementary Fig. 1i

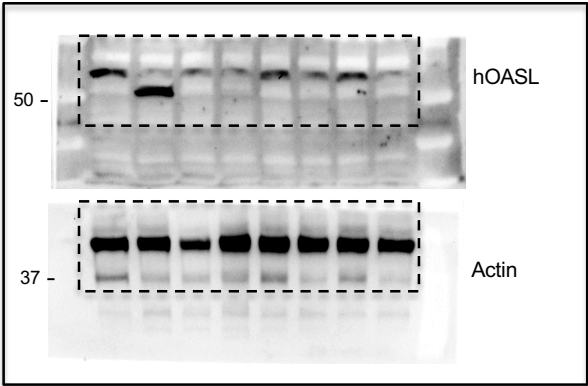

Supplement: Supplementary file 21 — Unprocessed western blot. [file 41556_2022_1039_MOESM21_ESM.pdf]

Supplementary Fig. 2c

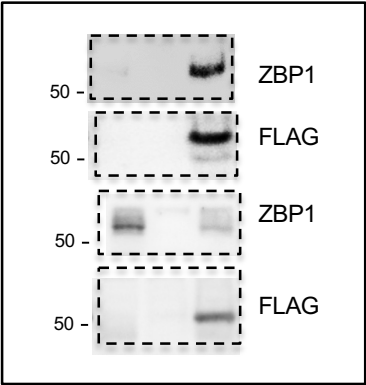

Supplement: Supplementary file 23 — Unprocessed western blot. [file 41556_2022_1039_MOESM23_ESM.pdf]
